# Supplementary material for: Biases in Demographic Modeling Affect Our Understanding of Recent Divergence
Source: Mol Biol Evol. 2021 Feb 24;38(7):2967–85. doi: 10.1093/molbev/msab047 (PMC8233503; doi:10.1093/molbev/msab047)
Supplement: msab047_Supplementary_Data [file msab047_supplementary_data.pdf]

Supplementary Information

Supplementary Table 1: Description of all models used to infer the demographic history from simulated and empirical data. When reconstructing the demographic history from empirical data, we also tested the same two-population demographic scenarios accounting for heterogeneous migration rates (2M), heterogeneous effective population size (2N) and both (2N2M).

Graphic representation	Demographic scenarios	Number of populations	Modifiers	Number of free parameters
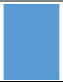	SNM<br>Standard Neutral Model	1	None	0
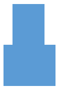	2EP<br>Two epochs model, one instantaneous change in N_e (expansion or contraction)	1	None	2
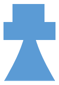	$3EP_B$<br>Three epochs model, one instantaneous change in N_e followed by bottle-growth	1	None	5
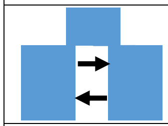	IM<br>Isolation with Migration	2	2M, 2N, 2M2N	Basic = 5<br>2N = 7<br>2M = 8<br>2M2N = 10
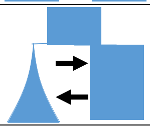	IM_B<br>Isolation with Migration. Bottleneck and growth in one population	2	2M, 2N, 2M2N	Basic = 6<br>2N = 8<br>2M = 9<br>2M2N = 11
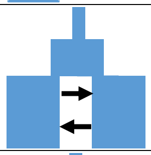	IM_{AE}<br>Isolation with Migration. One change in N_e in the ancestral population	2	2M, 2N, 2M2N	Basic= 7<br>2N = 9<br>2M = 10<br>2M2N = 13
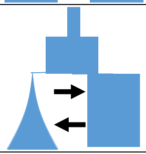	IM_{AEB}<br>Isolation with Migration. One change in N_e in the ancestral population, bottleneck and growth in a daughter population	2	2M, 2N, 2M2N	Basic = 8<br>2N = 10<br>2M = 11<br>2M2N = 13
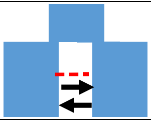	SC<br>Secondary Contact	2	2M, 2N, 2M2N	Basic = 6<br>2N = 8<br>2M = 9<br>2M2N = 11
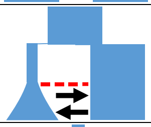	SC<br>Secondary Contact. Bottleneck and growth in one population	2	2M, 2N, 2M2N	Basic = 7<br>2N = 9<br>2M = 10<br>2M2N = 12
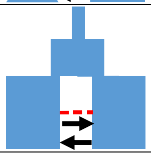	SC_{AE}<br>Secondary Contact. One change in N_e in the ancestral population	2	2M, 2N, 2M2N	Basic = 8<br>2N = 10<br>2M = 11<br>2M2N = 13
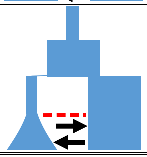	SC_{AEB}<br>Secondary Contact. One change in N_e in the ancestral population, bottleneck and growth in a daughter population	2	2M, 2N, 2M2N	Basic = 9<br>2N = 11<br>2M = 12<br>2M2N = 14

Supplementary Table 2: Location names, population codes, sample size (N) and geographic coordinates of all sampling locations of *S. maxixmus* used in this study.

Location	Population code	N	Latitude	Longitude	Reference
North Sea	NS	20	56.58° N	06.12° E	Nielsen et al. (2004)
Vendelsö	VD	27	57.17° N	12.06° E	Florin et al. (2007)
Öresund	OS	35	55.34° N	12.51° E	Florin et al. (2007)
Dabki	DAB	24	54.45° N	6.30° E	Florin et al. (2007)
Gdynia	GDY	23	54.45° N	8.30° E	Florin et al. (2007)
Gotland	GOT	20	57.32° N	18.57° E	Florin et al. (2007)
Gotska Station	GS	24	58.22 °N	19.15° E	Florin et al. (2007)

Supplementary Table 3: Parameter estimates of the two best models for the empirical study, $\pm$sd inferred using the Fisher Information Matrix /FIM) and the Godambe Information Matrix (FIM), the latter in brackets. AIC= Akaike Information Criterion, W_{AIC}= weight of evidence, $\Theta = 4N_{REF}\mu$, N_{AE}= N_e after population expansion, N_1= contemporary N_e in the North Sea, N_2= contemporary N_e in the Baltic Sea, s=N_e of the Baltic Sea at time of invasion as a proportion of N_1, T_{AE}= time of ancestral expansion, T_S = time of population divergence, T_{SC}= time of secondary contact, m_{12} = migration rates from the Baltic Sea to the North Sea, m_{21}= migration rates from the North Sea to the Baltic Sea, Bf= background factor, i.e. N_e of the part of the genome with reduced recombination/higher background selection as a proportion of N_e in the rest of the genome, Q= proportion of the genome with reduced N_e. Population sizes are given in units relative to N_{REF}. Times are given in units of $2N_{REF}$ generations, and need to be added backward in time (the real time of ancestral expansion for the IM_{AEB2n} model is T_{AE} + T_S). Migration rates are given in units $M_{ij} = 2N_{REFmij}$, where M_{ij} is the proportion of individuals in population i that is made up of migrants from population j at a given generation. For FIM we used the pruned SNPs dataset (1 SNP per 2b-RAD locus), for estimating uncertainties with the GIM we used all SNPs. Therefore, we have two Θ estimates (the one for the data set including all SNPs is in brackets, with its standard deviation estimated from the GIM.)

Model	*AIC*	W_{AIC}	Θ	N_{AE}	N_1	N_2	*s*	T_{AE}	T_S	T_{SC}	m_{12}	m_{21}	*Bf*	*Q*
IM_{AEB2N}	5536	0.66	2398 ±104 (2682 ±430)	1.68 ±0.069 (0.15)	2.78 ±0.56 (1.07)	2.77 ±0.73 (0.83)	0.0356 ±0.006 (0.004)	0.37 ±0.076 (0.065)	0.02 ±0.003 (0.004)	NA	4.66 ±2.35 (4.1)	28.15 ±2.01 (4.04)	0.1 ±0.013 (0.05)	0.22 ±0.03 (0.06)
SC_{AEB2N}	5537.5	0.31	2383 ±105 (2666 ±409)	1.675 ±0.07 (0.127)	2.898 ±0.62 (1.07)	2.75 ±0.7 (0.44)	0.054 ±0.023 (0.007)	0.38 ±0.08 (0.069)	0.003 ±0.003 (0.002)	0.017 ±0.0037 (0.0027)	4.53 ±2.54 (3.22)	28.15 ±2.26 (1.84)	0.1 ±0.012 (0.049	0.22 ±0.03 (0.06)

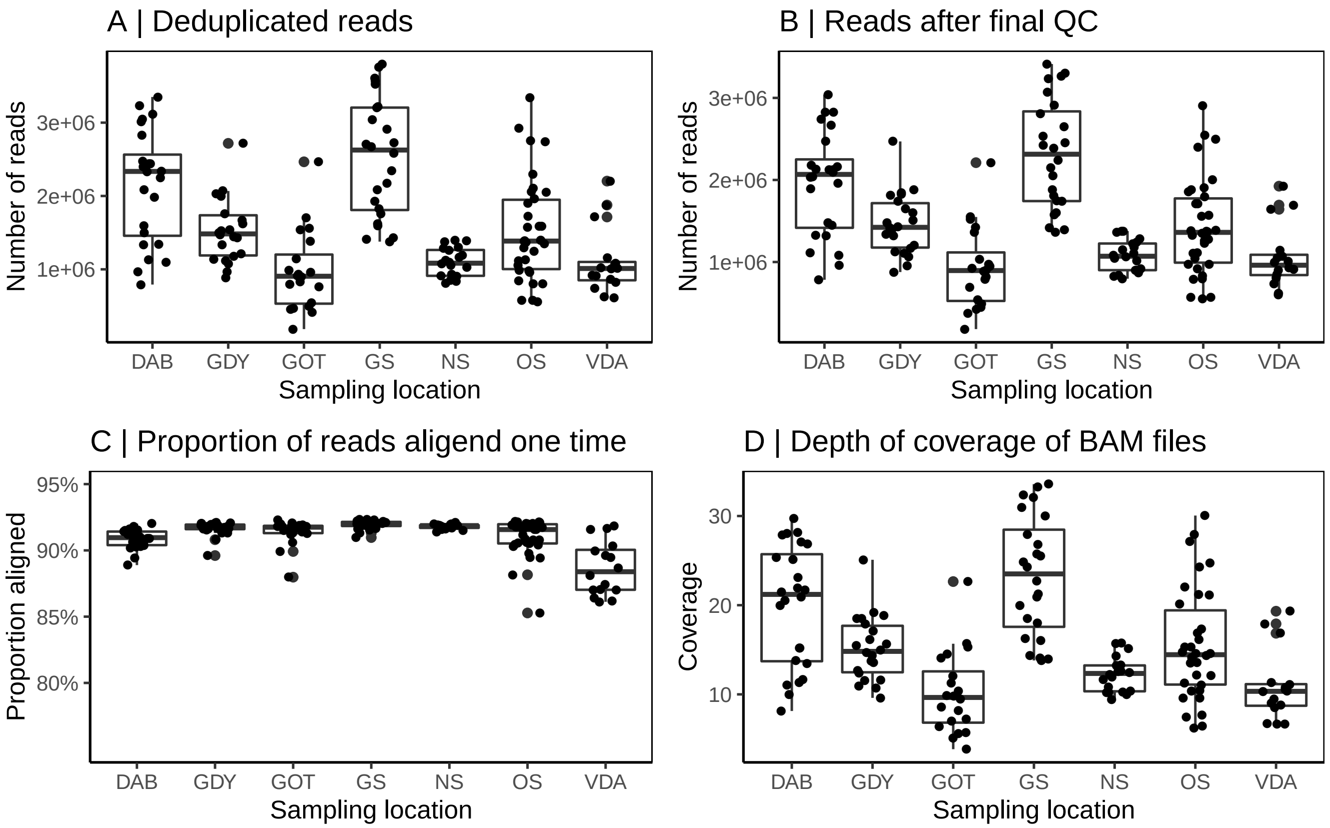

Supplementary Fig. 1: summary statistics from 2b-RAD data. Number of deduplicated reads (**A**) reads after quality check (**B**), proportion of reads which mapped one time to the reference genome (**C**) and mean per individual depth of coverage calculated from BAM files (**D**).

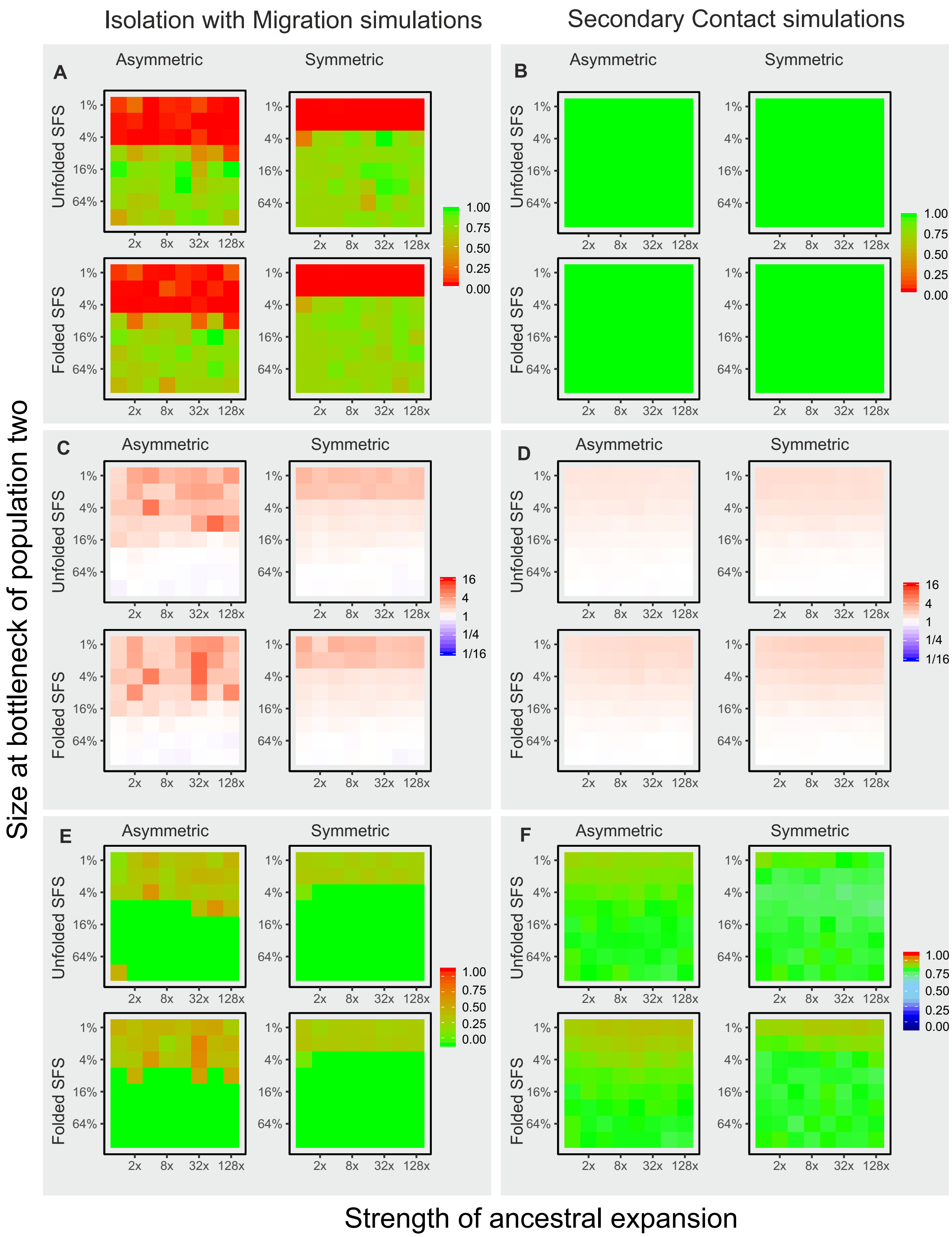

Supplementary Fig. 2: Model choice and parameter misestimation for all eight divergence models for all simulations of the larger data sets (1 million loci). Left panels **A, C & C** show results from simulations with constant migration (IM), right panels **B, D & F** show results from simulation with a period of strict isolation (SC). Within each panel, results are shown for simulations with symmetric and asymmetric migration, and for model estimates using the folded or unfolded jAFS. Within each panel, each graph represent the values for all 64 simulations as per Fig. 1. Panel **A** and **B** show weight of evidence for the correct gene flow scenario (0-1). **C & D** show misestemation of the parameter T_S (T_S estimate /T_S of simulation). Panels **E** and **F** show the estimated proportion of the divergence time for which the model inferred strict isolation (green represent the correct time, i.e. 0 for IM model and 0.75 for SC models).

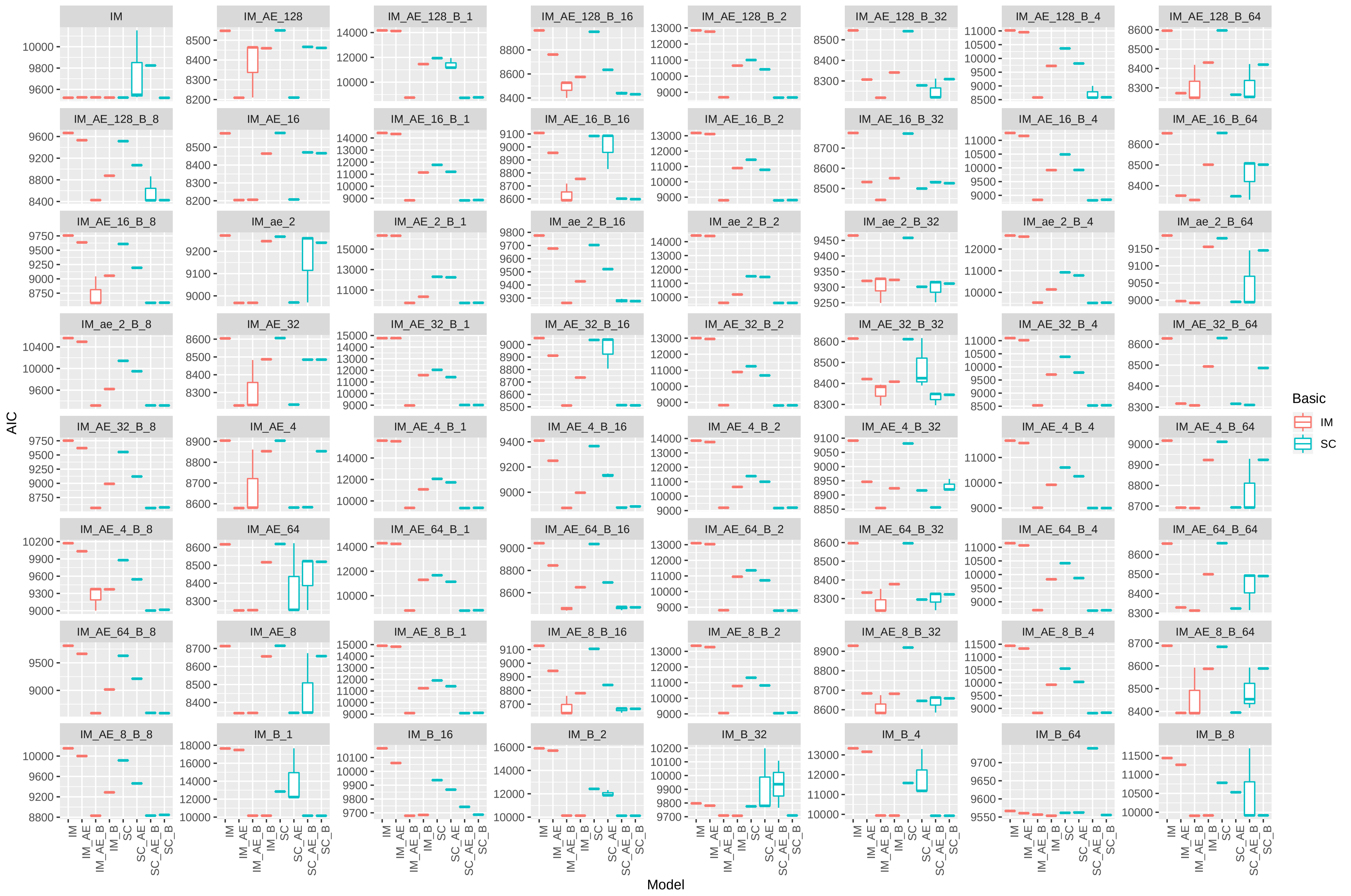

Supplementary Fig. 3: Boxplot showing AIC estimates for IM and SC models for the unfolded jAFS of 64 simulations (1 million loci) under the asymmetric IM model. The best three replicates for each model/simulation combination are plotted. Note the very narrow boxplots showing models have converged to the same likelihhods, with a few exception.

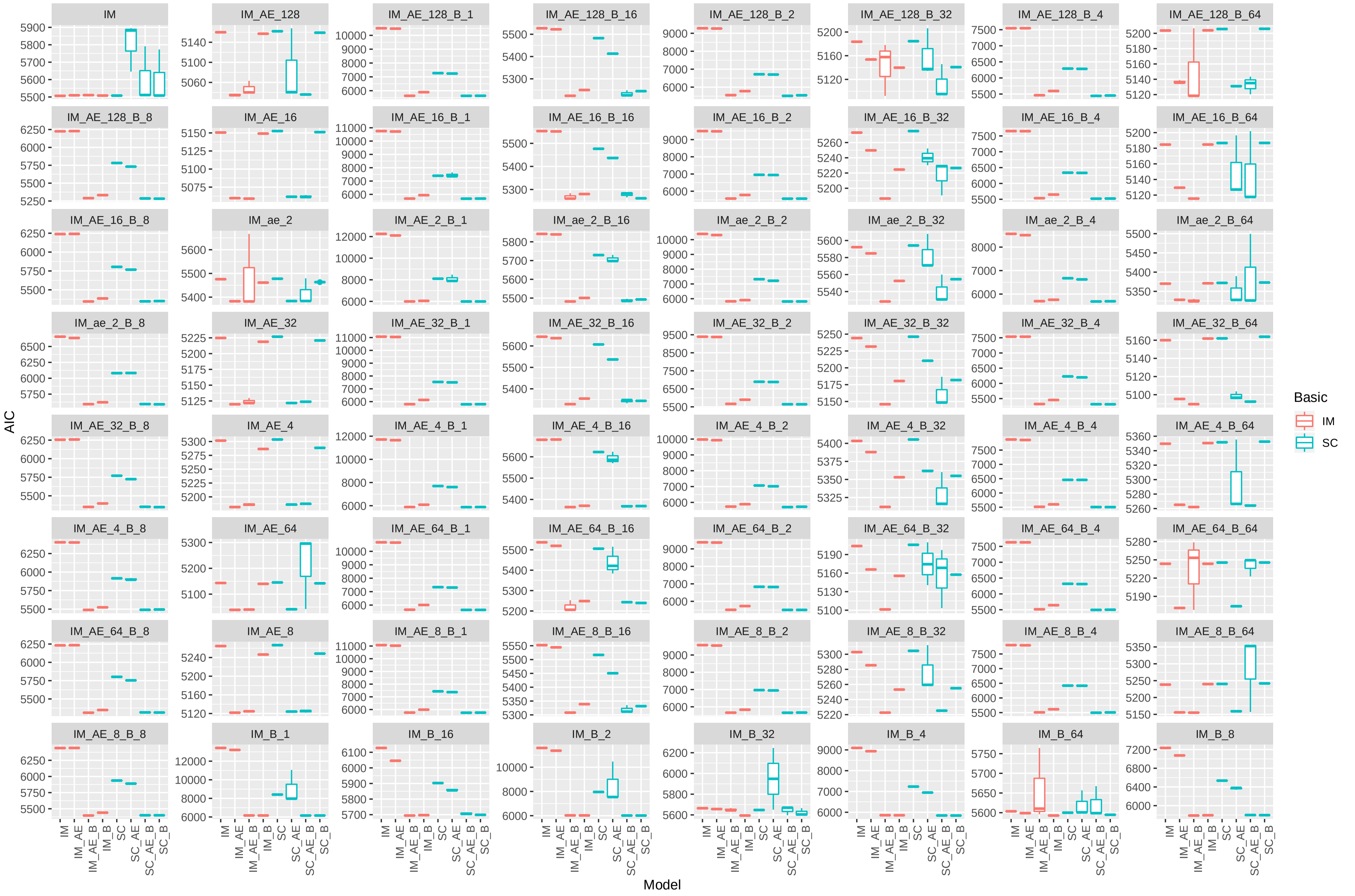

Supplementary Fig. 4: Boxplot showing AIC estimates for IM and SC models for the folded jAFS of 64 simulations (1 million loci) under the asymmetric IM model. The best three replicates for each model/simulation combination are plotted. Note the very narrow boxplots showing models have converged to the same likelihhods, with a few exception.

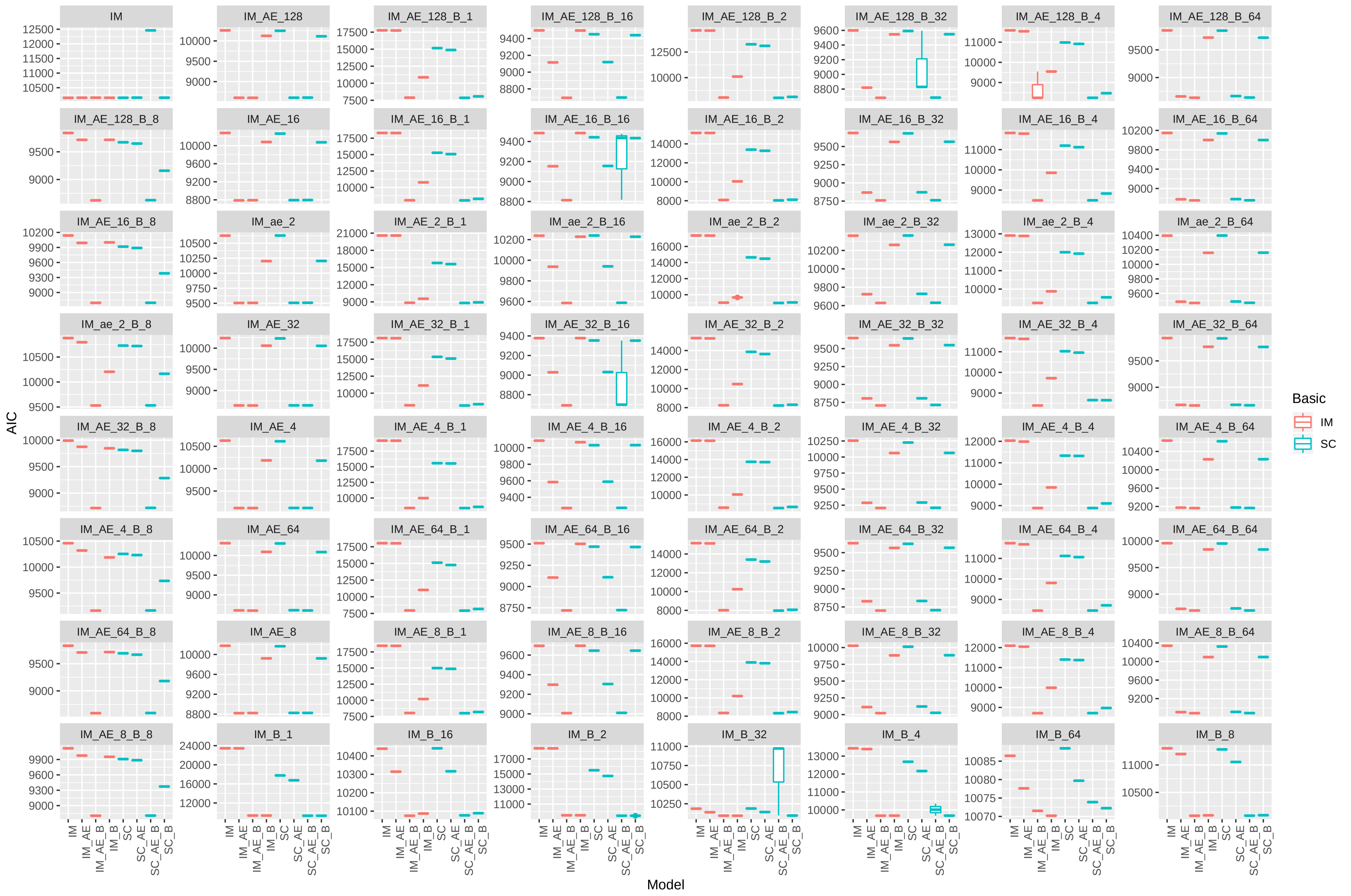

Supplementary Fig. 5: Boxplot showing AIC estimates for IM and SC models for the unfolded jAFS of 64 simulations (1 million loci) under the symmetric IM model. The best three replicates for each model/simulation combination are plotted. Note the very narrow boxplots showing models have converged to the same likelihhods, with a few exception.

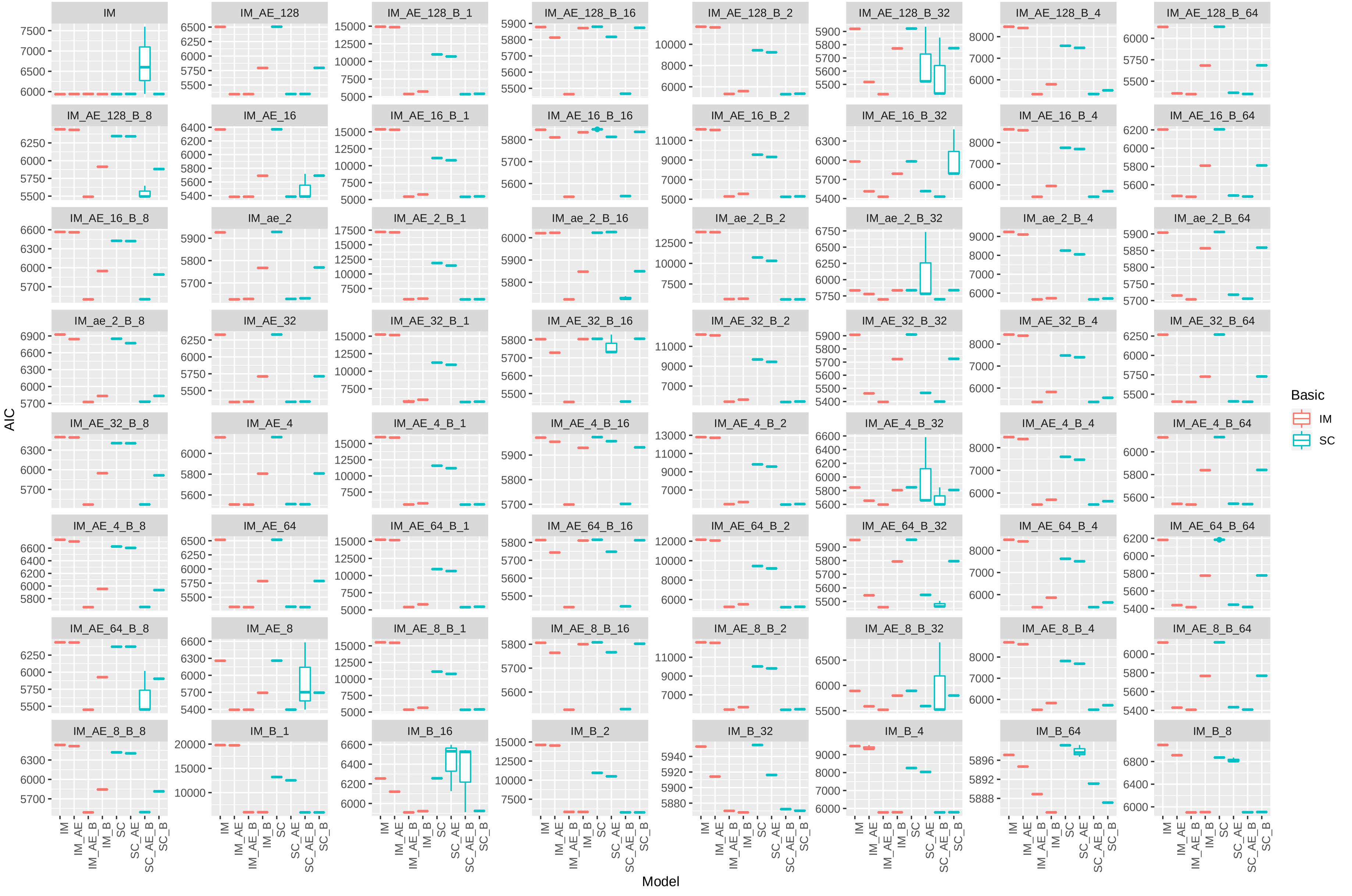

Supplementary Fig. 6: Boxplot showing AIC estimates for IM and SC models for the folded jAFS of 64 simulations (1 million loci) under the symmetric IM model. The best three replicates for each model/simulation combination are plotted. Note the very narrow boxplots showing models have converged to the same likelihoods, with a few exception.

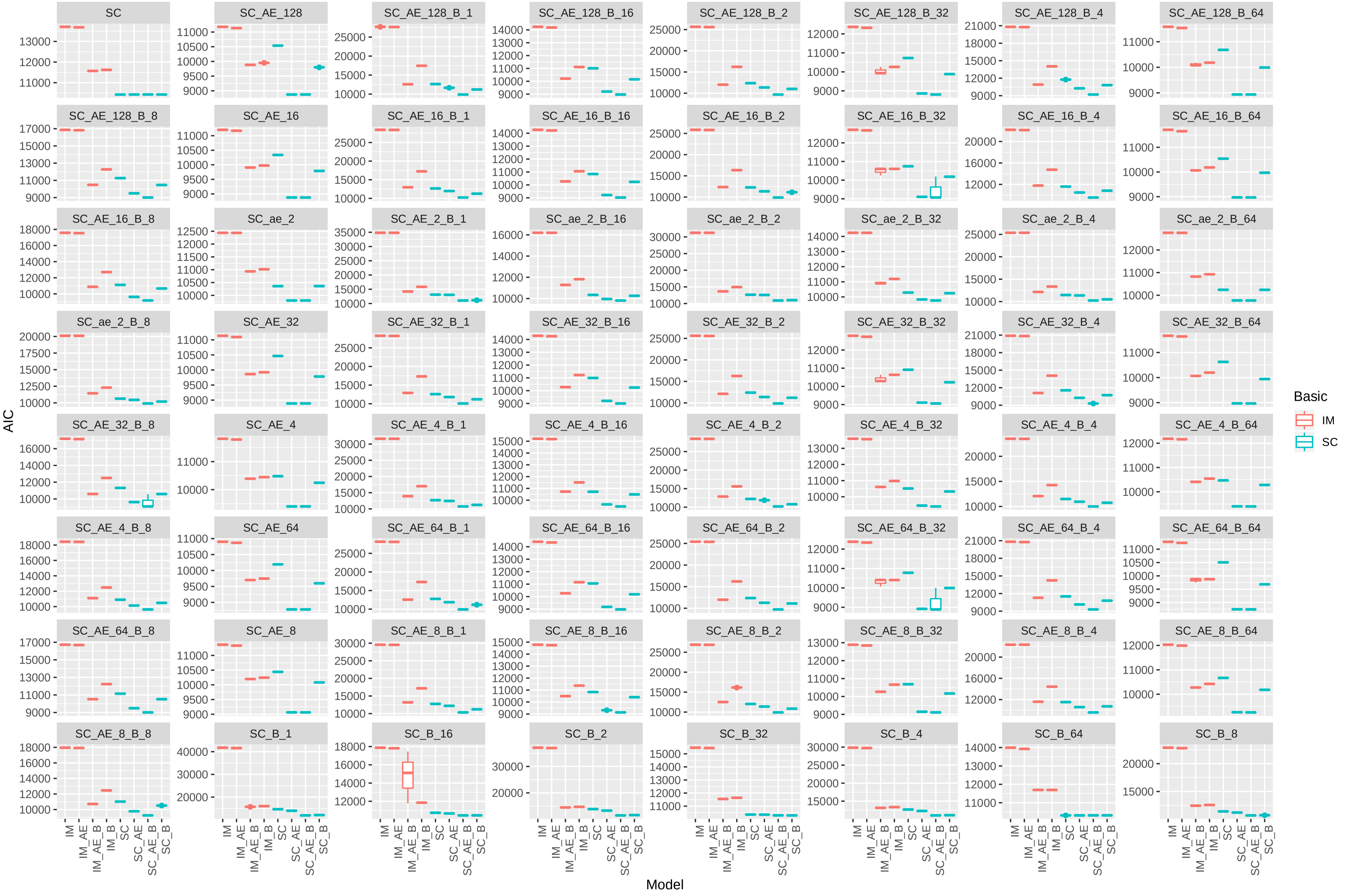

Supplementary Fig. 7: Boxplot showing AIC estimates for IM and SC models for the unfolded jAFS of 64 simulations (1 million loci) under the asymmetric SC model. The best three replicates for each model/simulation combination are plotted. Note the very narrow boxplots showing models have converged to the same likelihhods, with a few exception.

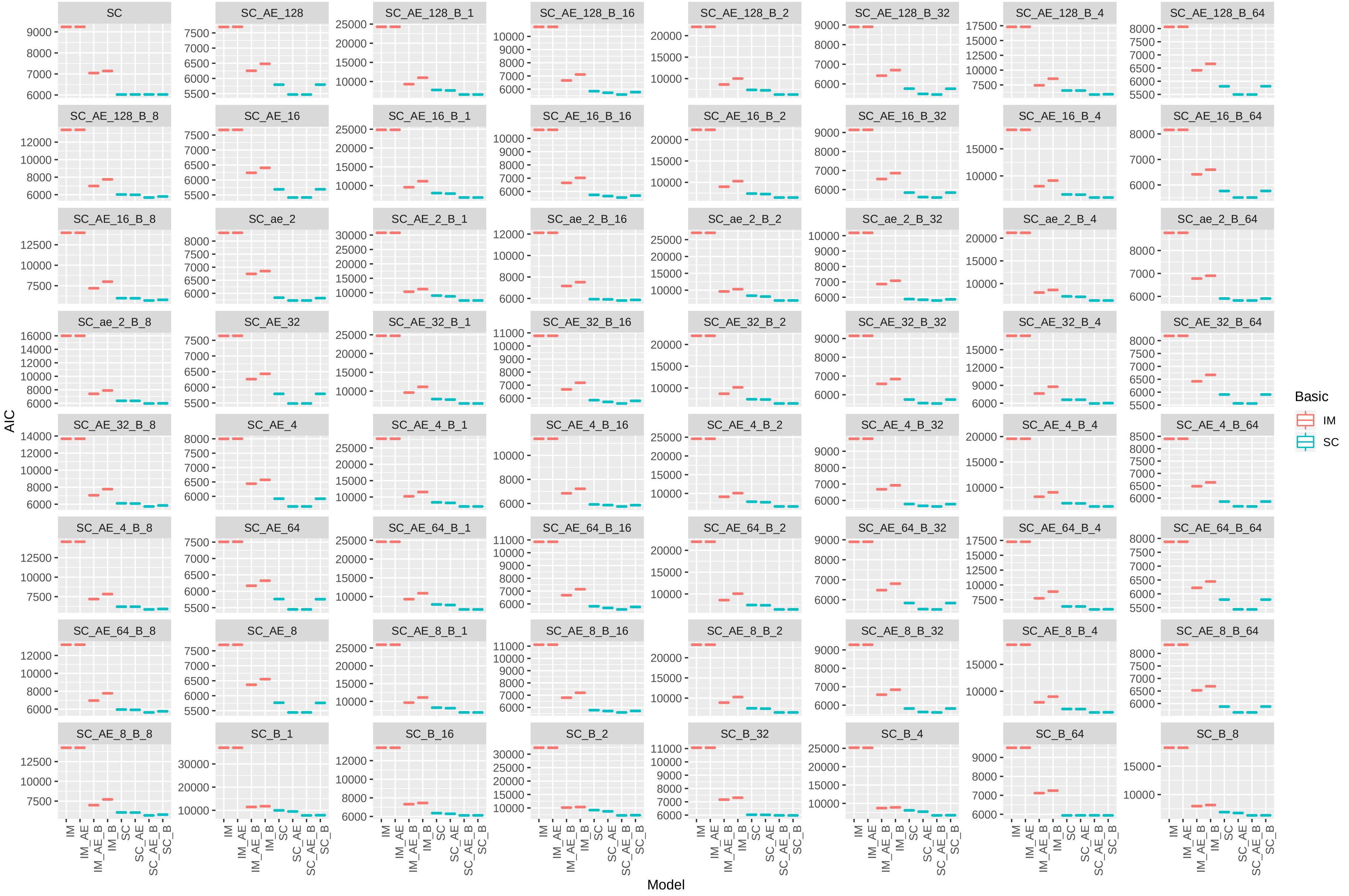

Supplementary Fig. 8: Boxplot showing AIC estimates for IM and SC models for the folded jAFS of 64 simulations (1 million loci) under the asymmetric SC model. The best three replicates for each model/simulation combination are plotted. Note the very narrow boxplots showing models have converged to the same likelihods, with a few exception.

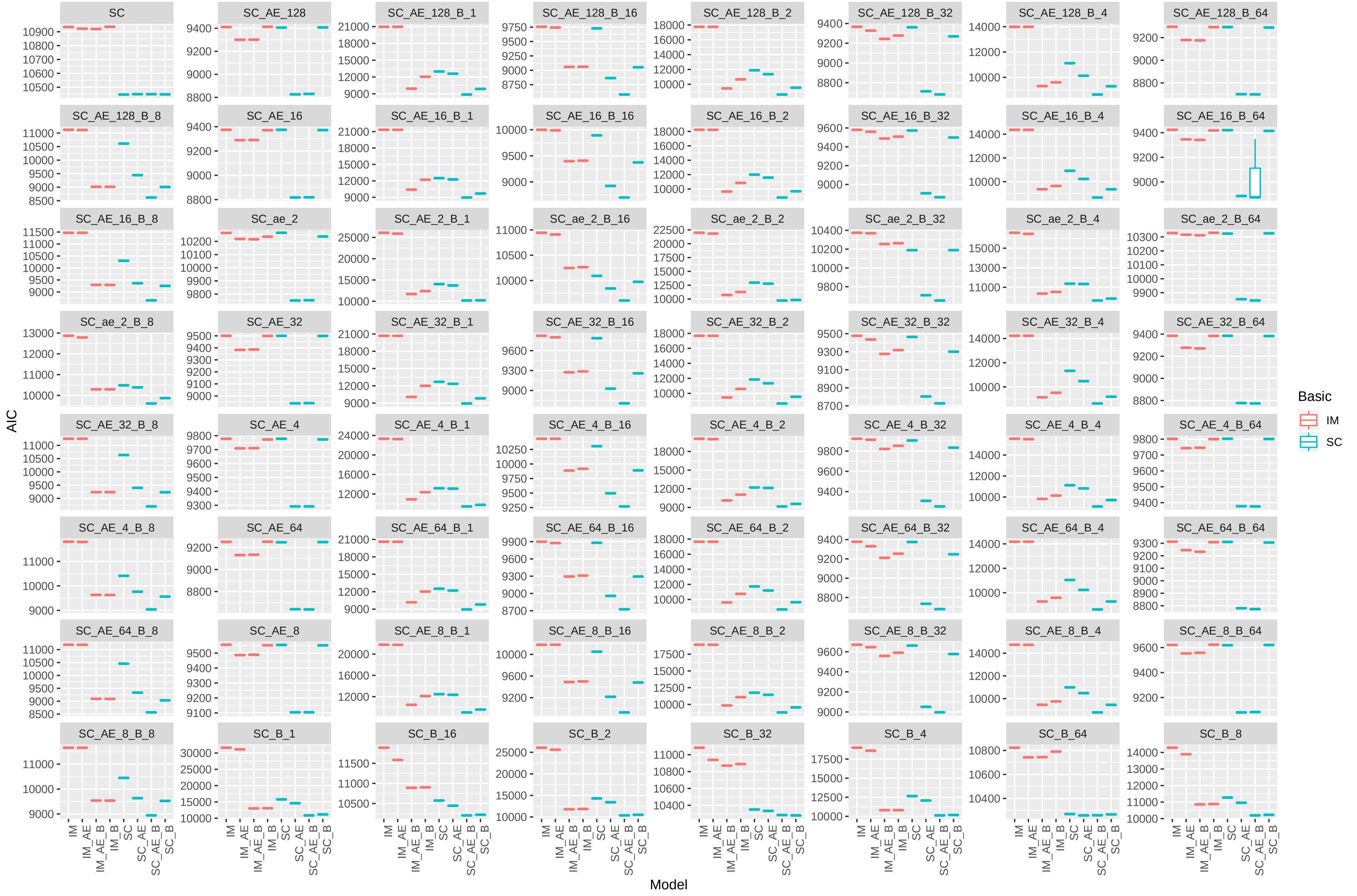

Supplementary Fig. 9: Boxplot showing AIC estimates for IM and SC models for the unfolded jAFS of 64 simulations (1 million loci) under the symmetric SC model. The best three replicates for each model/simulation combination are plotted. Note the very narrow boxplots showing models have converged to the same likelihhods, with a few exception.

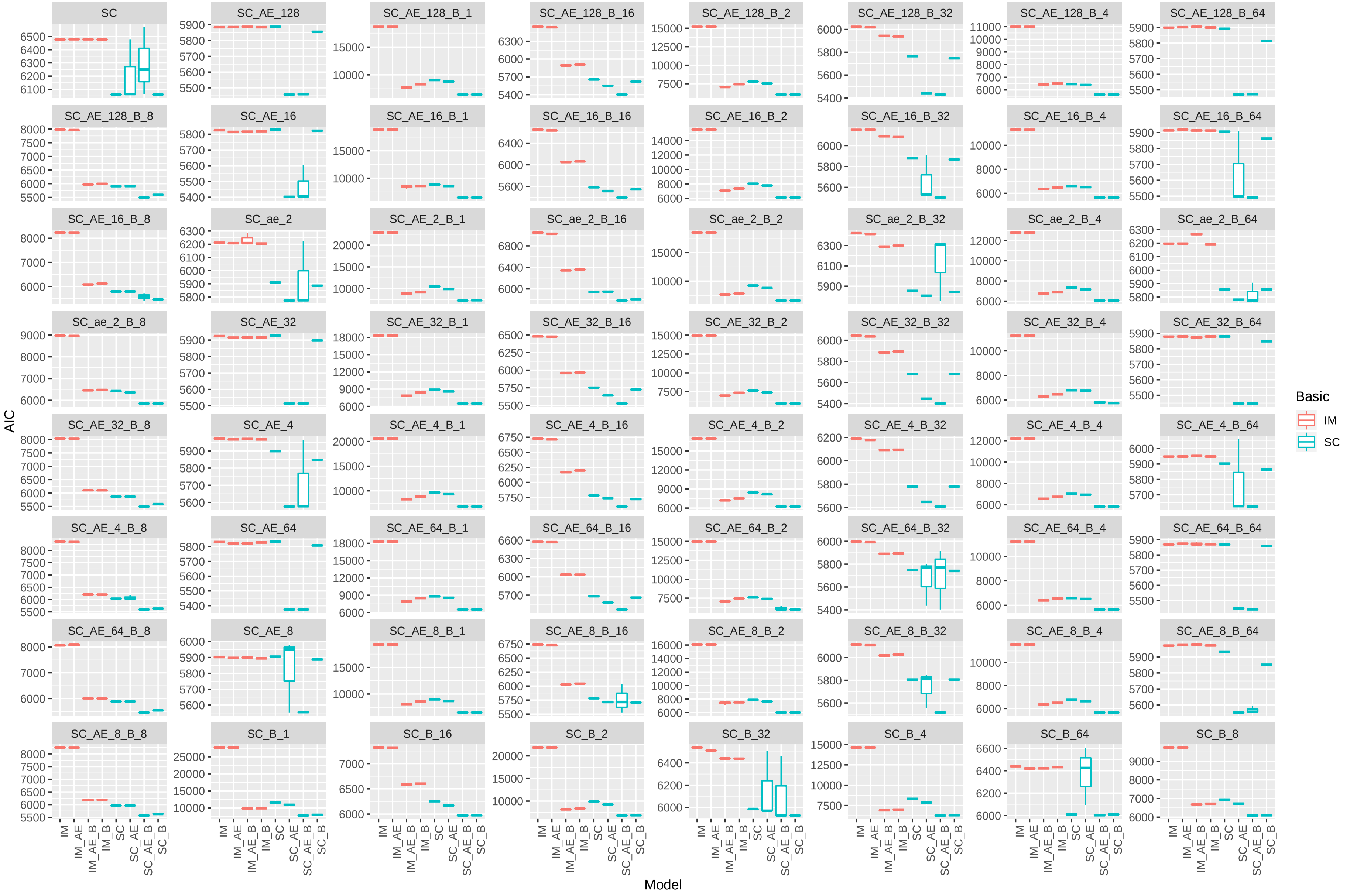

Supplementary Fig. 10: Boxplot showing AIC estimates for IM and SC models for the folded jAFS of 64 simulations (1 million loci) under the symmetric SC model. The best three replicates for each model/simulation combination are plotted. Note the very narrow boxplots showing models have converged to the same likelihhods, with a few exception.

Supplementary Fig. 11: misestimation of T_S. Columns in the grids group by simulation Ancestral Expansion (from 1, i.e. no expansion, to 128 x). The x axes represent the size of population two (N_2) at time of split (T_S) as a proportion of current N_2. Rows of the plot grids group by gene flow scenario of the simulation (IM and SC) and by whether the unfolded or folded spectrum was used for inference. Panels A and B show T_S misestimation when the basic IM and SC models are used for inference, and under a symmetric and asymmetric migration scenario respectively. Panels C and D show (T_S) misestimation when the full 8 models (IM,SC, IMAE, SC_{AE}, IM_B, SC_B, IM_{AEB}, SC_{AEB} are used. For each model, T_S misestimation was calculated as $T_S^{model}/T_S^{simulation}$.

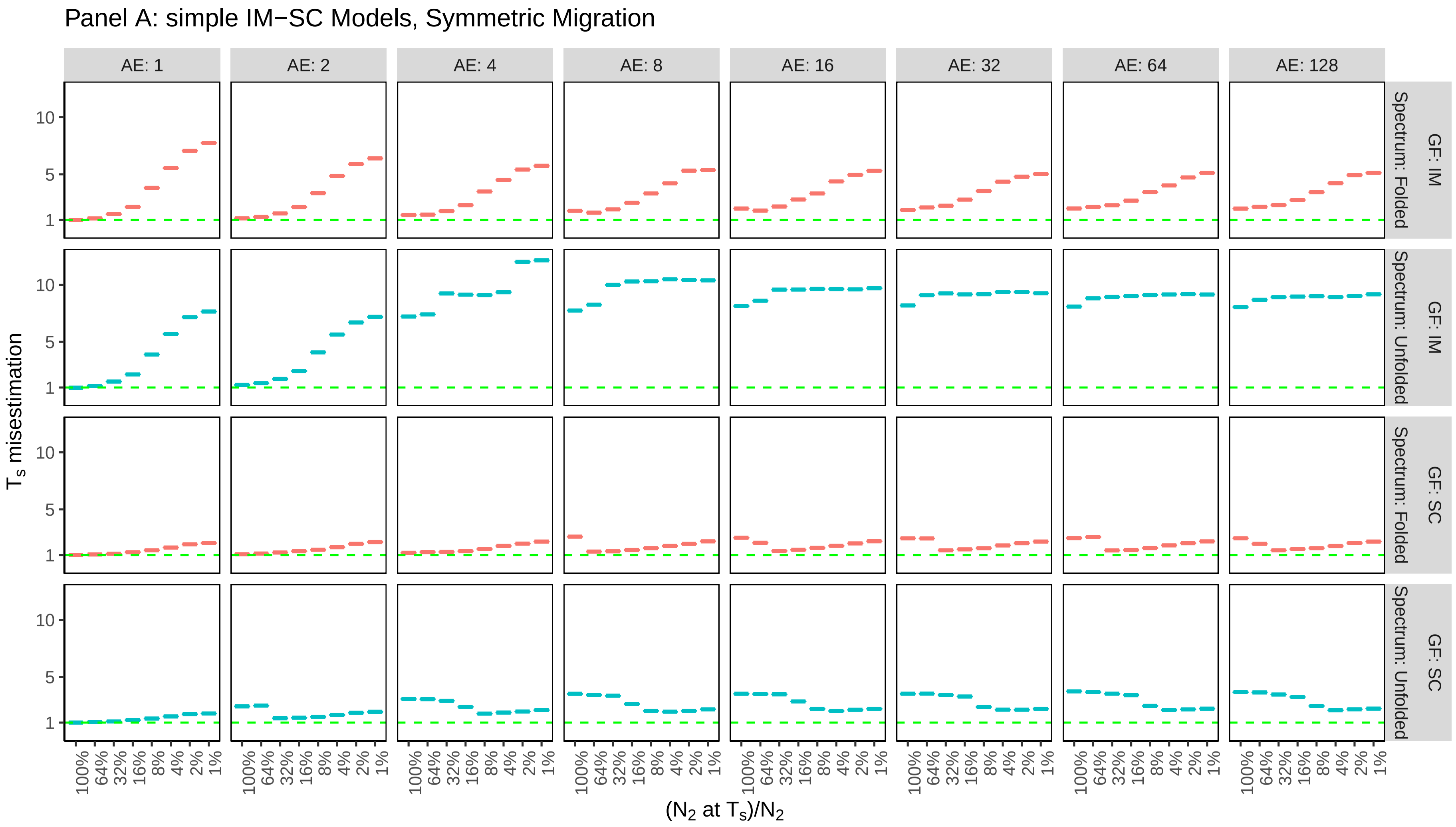

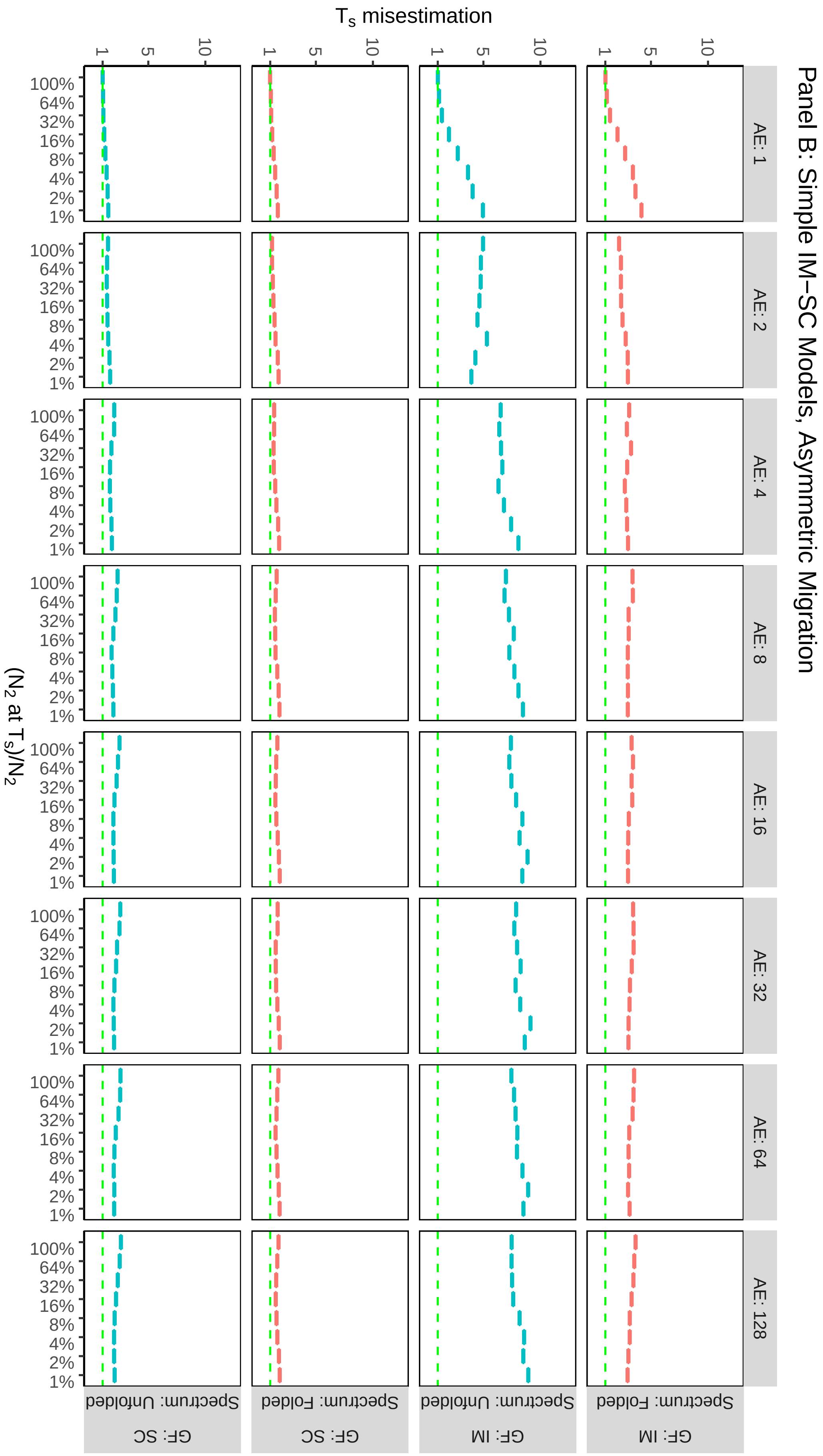
Panel B: Simple IM–SC Models, Asymmetric Migration
Ts misestimation
1
5
10
AE: 1
AE: 2
AE: 4
AE: 8
AE: 16
AE: 32
AE: 64
AE: 128
100%
64%
32%
16%
8%
4%
2%
1%
(N2 at Ts)/N2
Spectrum: Folded
GF: IM
Spectrum: Unfolded
GF: IM
Spectrum: Folded
GF: SC
Spectrum: Unfolded
GF: SC

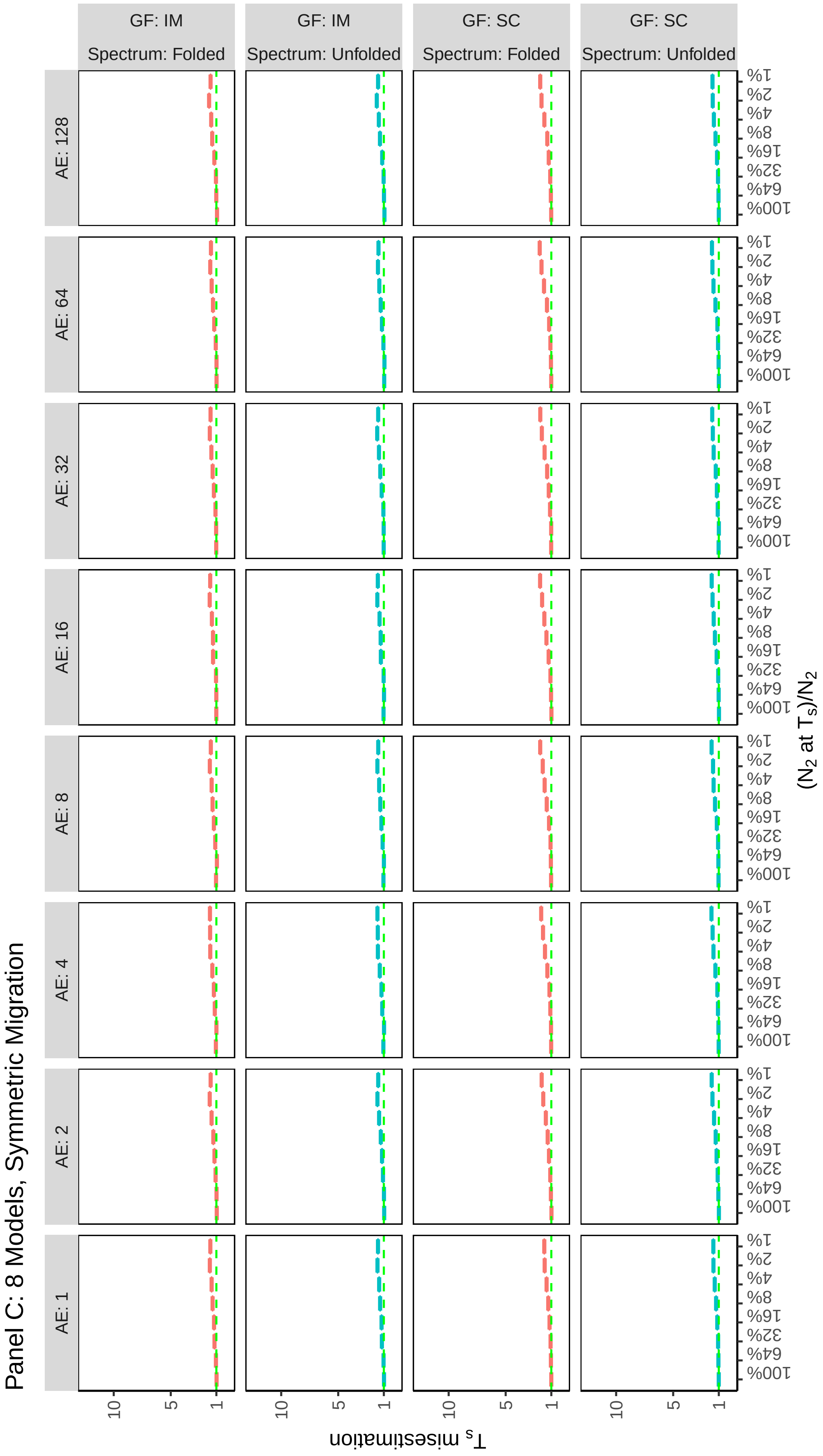

Panel C: 8 Models, Symmetric Migration
GF: IM
Spectrum: Folded
GF: IM
Spectrum: Unfolded
GF: SC
Spectrum: Folded
GF: SC
Spectrum: Unfolded
AE: 128
AE: 64
AE: 32
AE: 16
AE: 8
AE: 4
AE: 2
AE: 1
1%
2%
4%
8%
16%
32%
64%
100%
$(N_2$ at $T_S)/N_2$
10
5
1
T_S misestimation

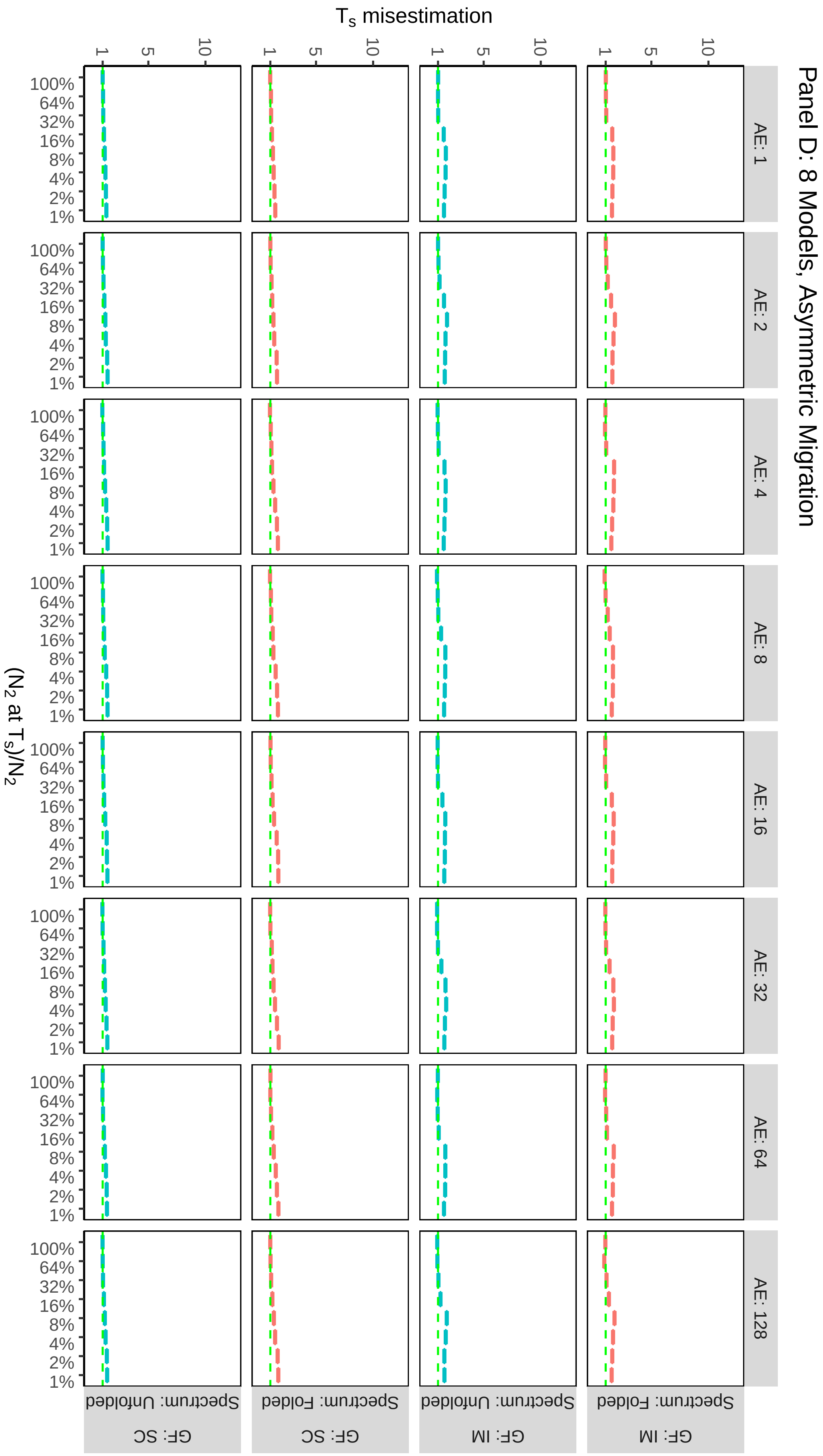

Panel D: 8 Models, Asymmetric Migration
T_S misestimation
$(N_2 \text{ at } T_S)/N_2$
AE: 1
AE: 2
AE: 4
AE: 8
AE: 16
AE: 32
AE: 64
AE: 128
GF: IM
GF: SC
Spectrum: Folded
Spectrum: Unfolded
1
5
10
100%
64%
32%
16%
8%
4%
2%
1%

Supplementary Fig. 12: Inferred proportion of SI during divergence by the best model. Columns and rows in the grids group by simulations as for Supplementary Fig. 11. Green dotted lines represent the true proportion of SI for the simulation scenarios. For each simulation the value reported is the value from the model replicate with the lowest AIC.

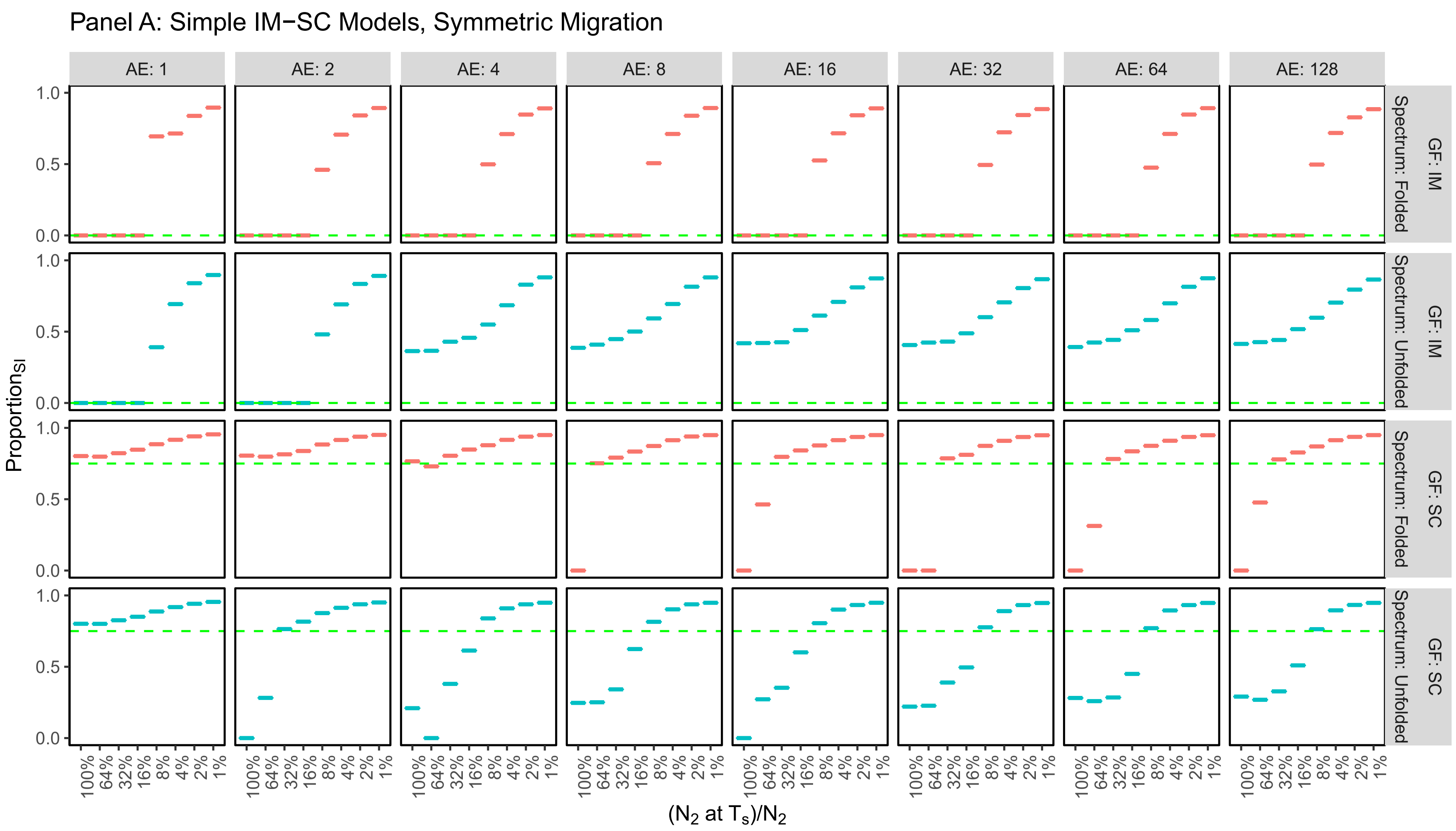

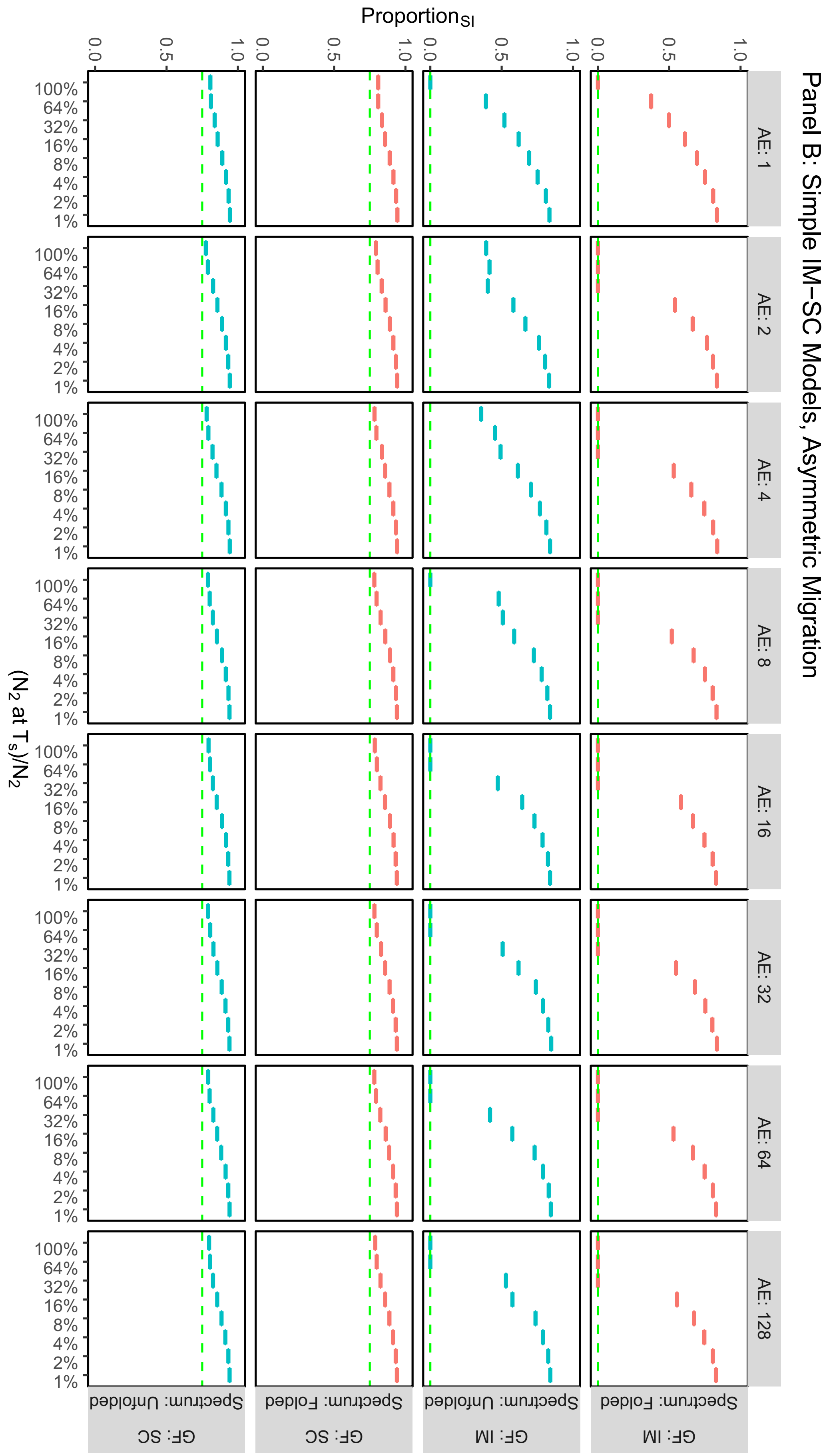

Panel B: Simple IM–SC Models, Asymmetric Migration
ProportionSI
AE: 1
AE: 2
AE: 4
AE: 8
AE: 16
AE: 32
AE: 64
AE: 128
GF: IM
GF: SC
Spectrum: Folded
Spectrum: Unfolded
0.0
0.5
1.0
100%
64%
32%
16%
8%
4%
2%
1%
(N2 at Ts)/N2

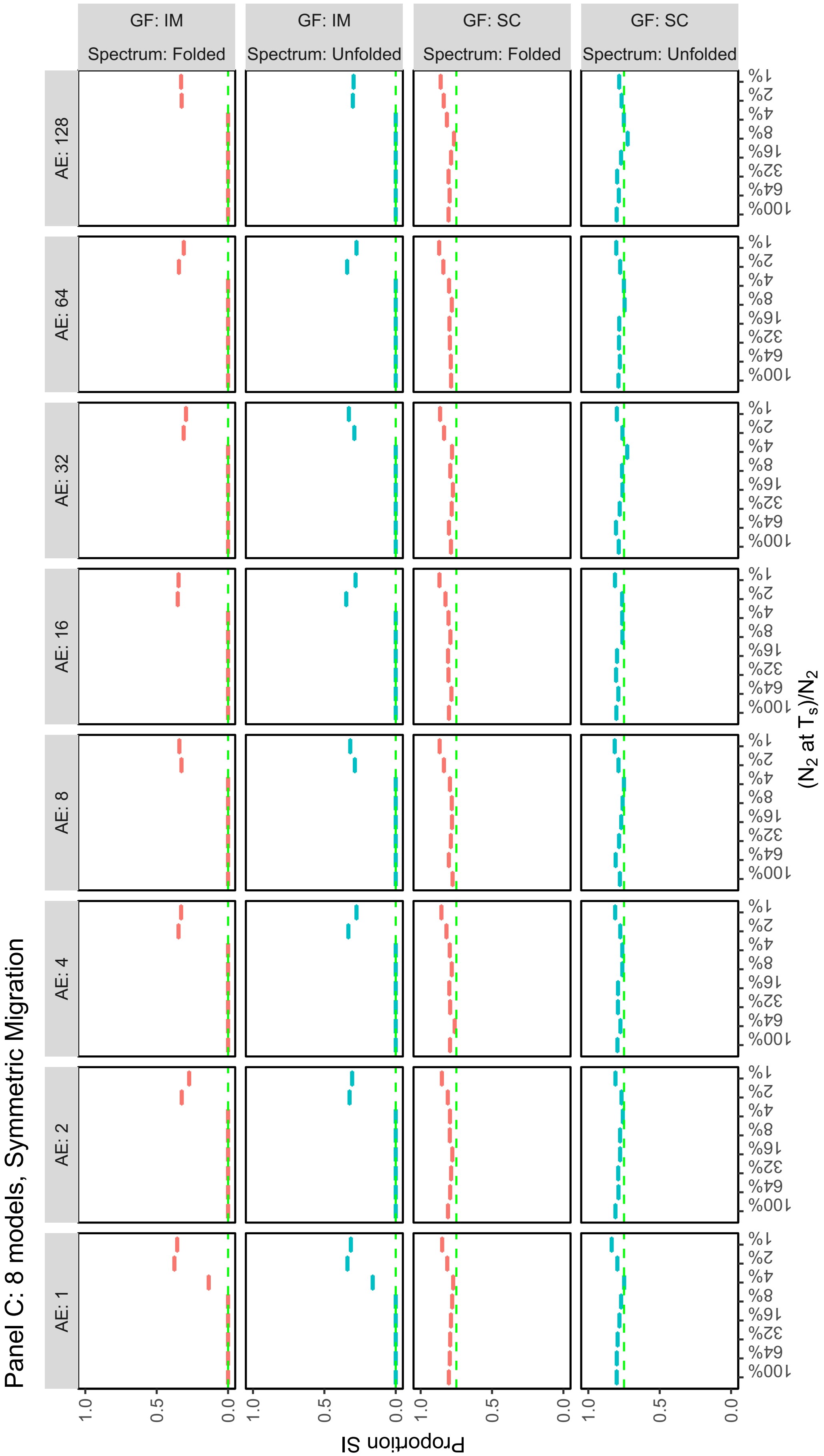

Panel C: 8 models, Symmetric Migration
GF: IM
Spectrum: Folded
GF: IM
Spectrum: Unfolded
GF: SC
Spectrum: Folded
GF: SC
Spectrum: Unfolded
AE: 128
AE: 64
AE: 32
AE: 16
AE: 8
AE: 4
AE: 2
AE: 1
1%
2%
4%
8%
16%
32%
64%
100%
1.0
0.5
0.0
Proportion SI
(N2 at Ts)/N2

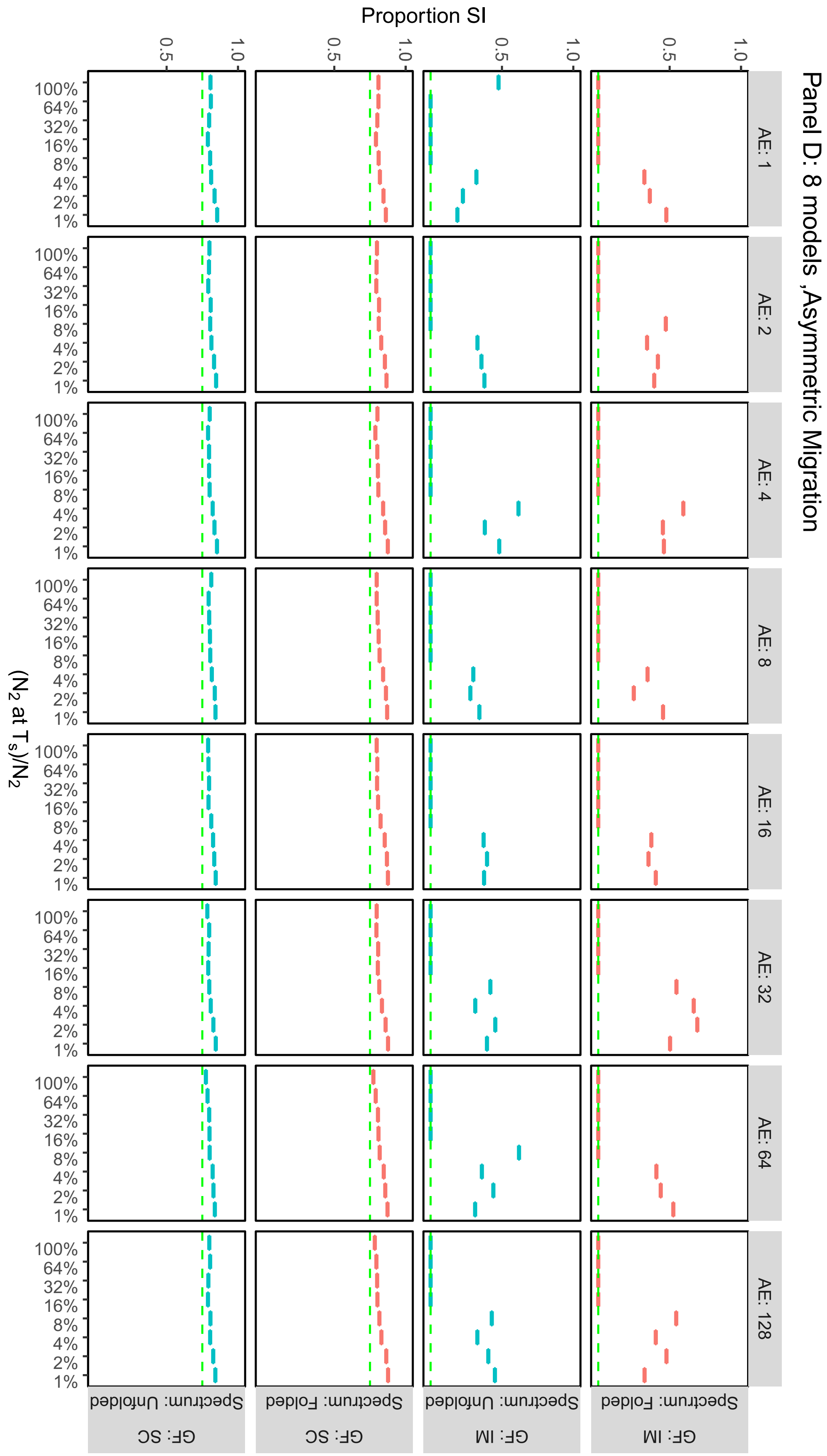

Panel D: 8 models ,Asymmetric Migration
Proportion SI
AE: 1
AE: 2
AE: 4
AE: 8
AE: 16
AE: 32
AE: 64
AE: 128
GF: IM
Spectrum: Folded
GF: IM
Spectrum: Unfolded
GF: SC
Spectrum: Folded
GF: SC
Spectrum: Unfolded
$(N_2$ at $T_s)/N_2$
100%
64%
32%
16%
8%
4%
2%
1%
0.5
1.0

Supplementary Fig. 13: misestimation of N_{ANC}. Columns in the grids group by simulation Ancestral Expansion (from 1, i.e. no expansion, to 128 x). The x axes represent the size of population 2 (N_2) at time of split (T_S) as a proportion of current (N_2). Rows of the plot grids group by gene flow scenario of the simulation (IM and SC) and by whether the unfolded or folded spectrum was used for inference. Panels **A** and **B** show N_{ANC} misestimation when the basic IM and SC models are used for inference, and under a symmetric and asymmetric migration scenario respectively. Panels **C** and **D** show N_{ANC} misestimation when the full 8 models (IM,SC,IM_{AE}, SC_{AE}, IM_B, SC_B, IM_{AEB}, SC_{AEB} are used. For each model,N_{ANC} misestimation was calculated as N_{ANC}model/ N_{ANC} simulation.

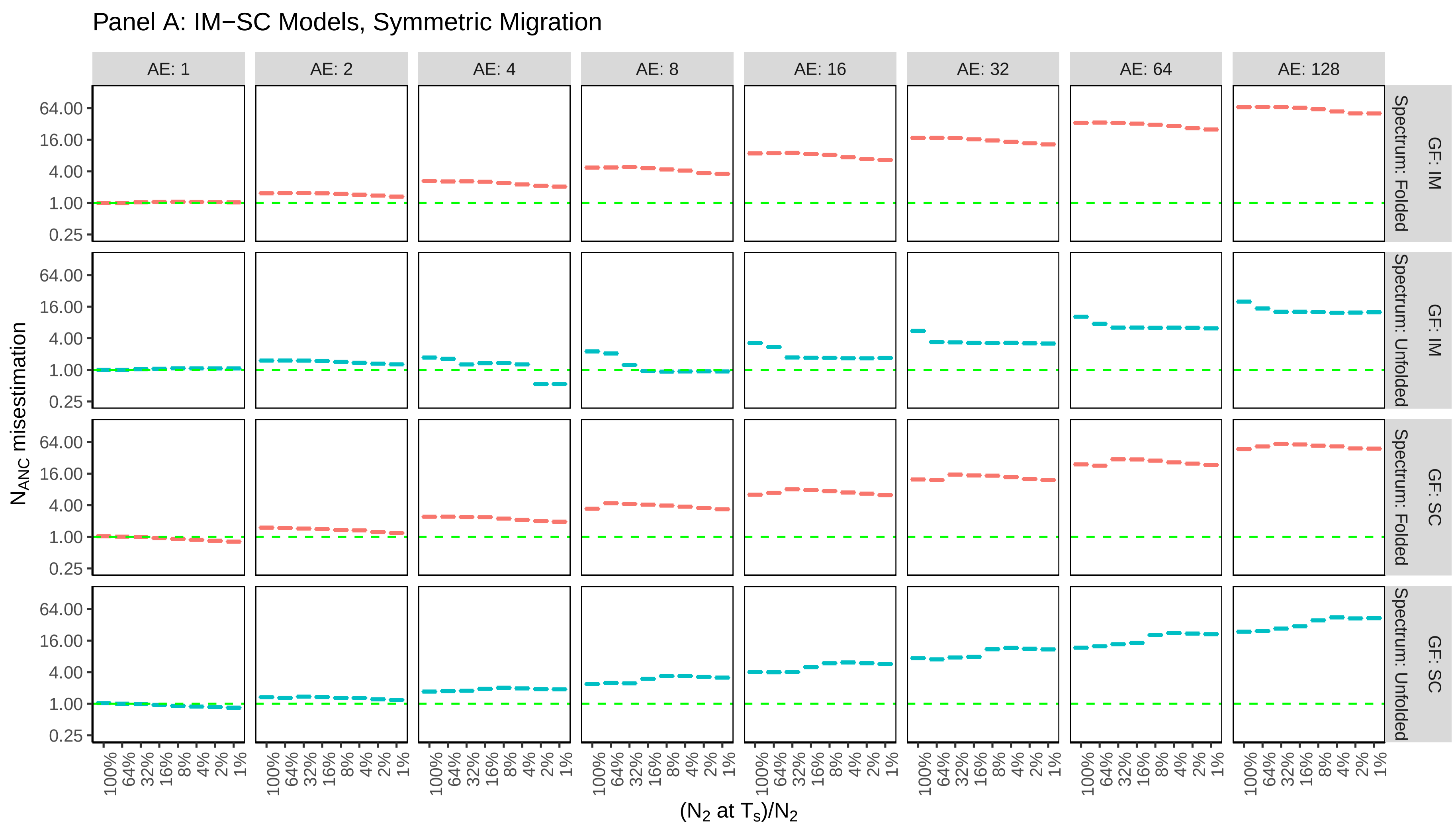

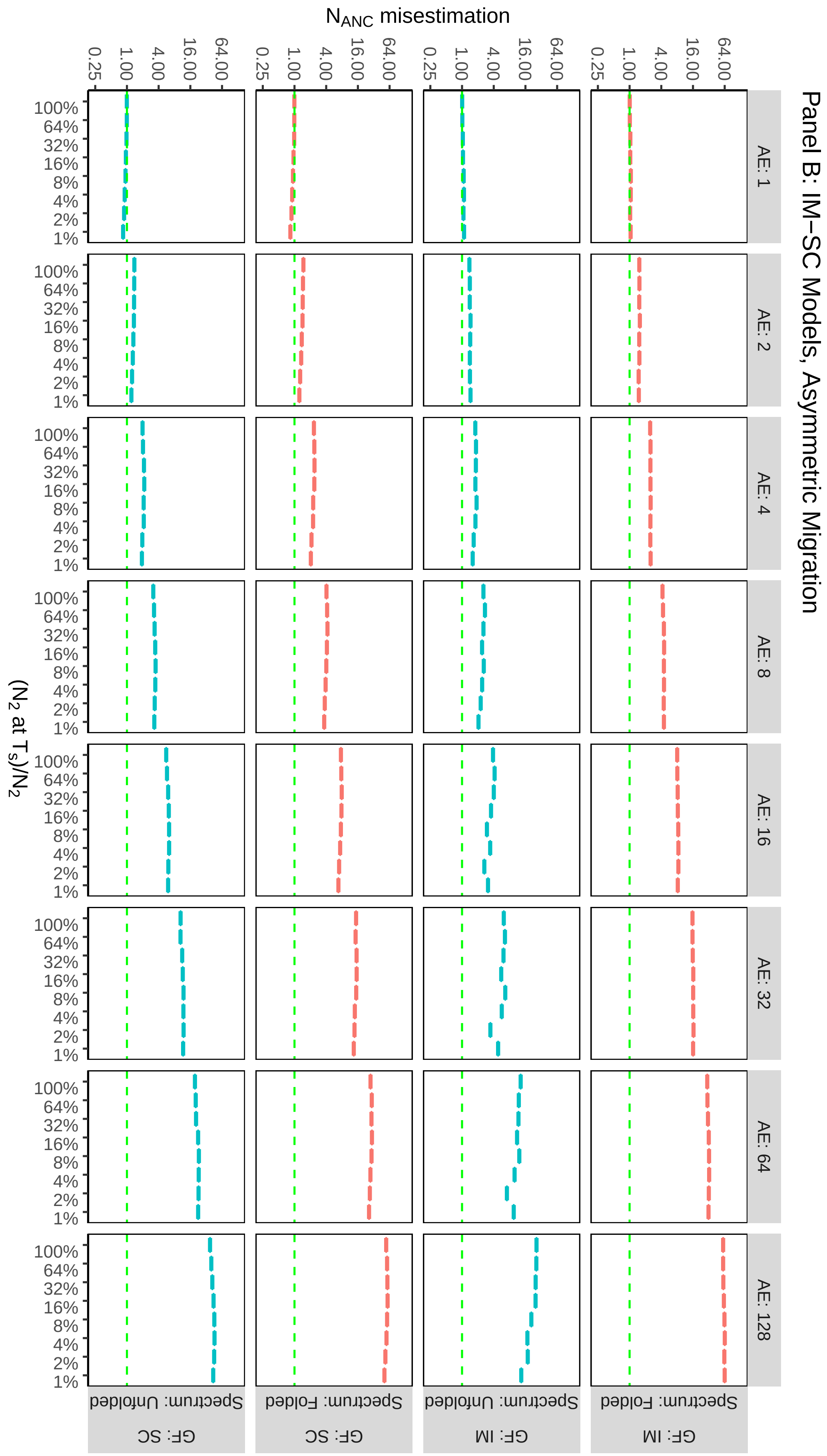
Panel B: IM–SC Models, Asymmetric Migration
N_ANC misestimation
0.25
1.00
4.00
16.00
64.00
(N2 at Ts)/N2
100%
64%
32%
16%
8%
4%
2%
1%
AE: 1
AE: 2
AE: 4
AE: 8
AE: 16
AE: 32
AE: 64
AE: 128
Spectrum: Folded
Spectrum: Unfolded
GF: IM
GF: SC

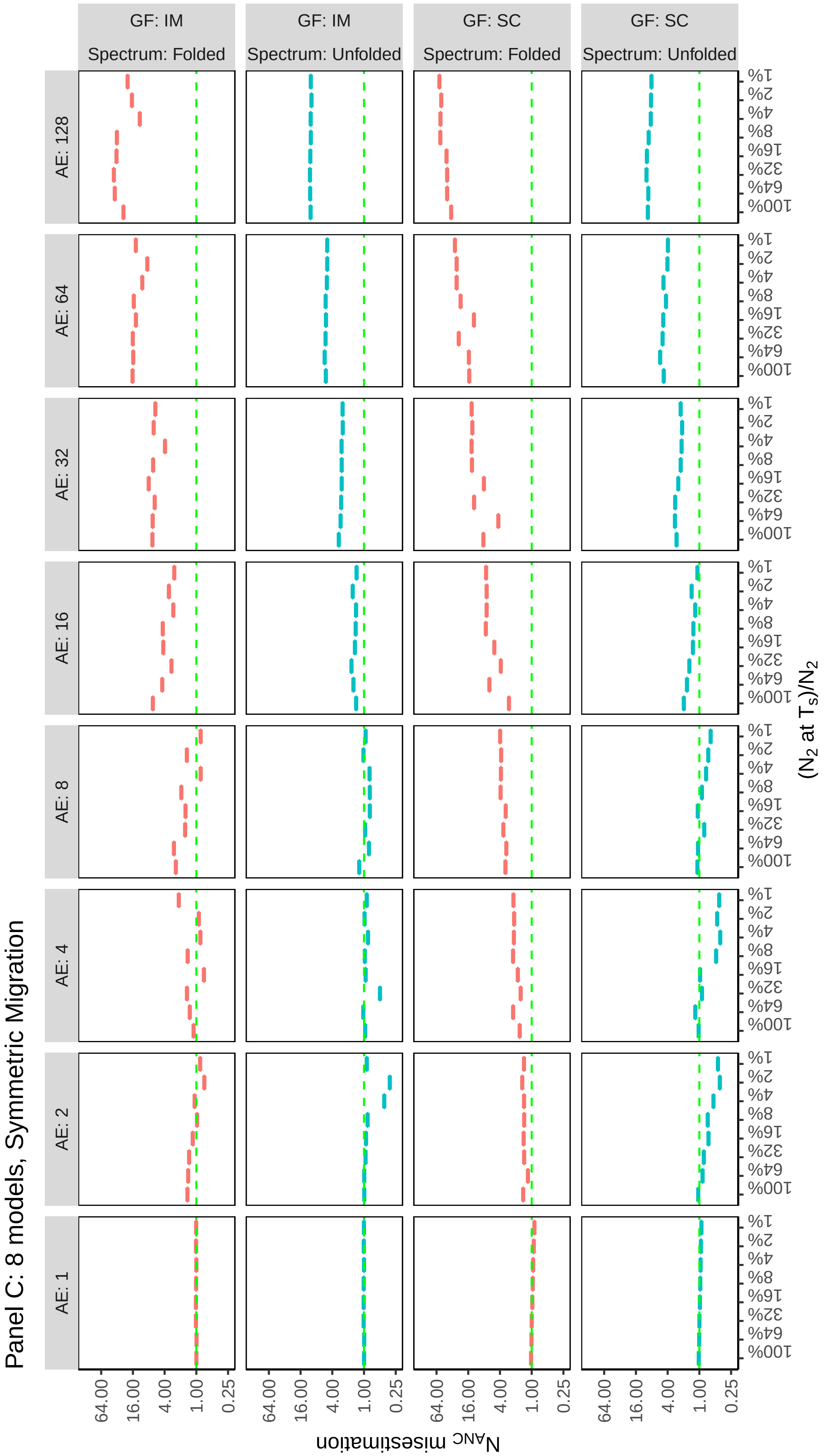
Panel C: 8 models, Symmetric Migration
GF: IM
Spectrum: Folded
GF: IM
Spectrum: Unfolded
GF: SC
Spectrum: Folded
GF: SC
Spectrum: Unfolded
AE: 128
AE: 64
AE: 32
AE: 16
AE: 8
AE: 4
AE: 2
AE: 1
1%
2%
4%
8%
16%
32%
64%
100%
(N2 at TS)/N2
64.00
16.00
4.00
1.00
0.25
NANC misestimation

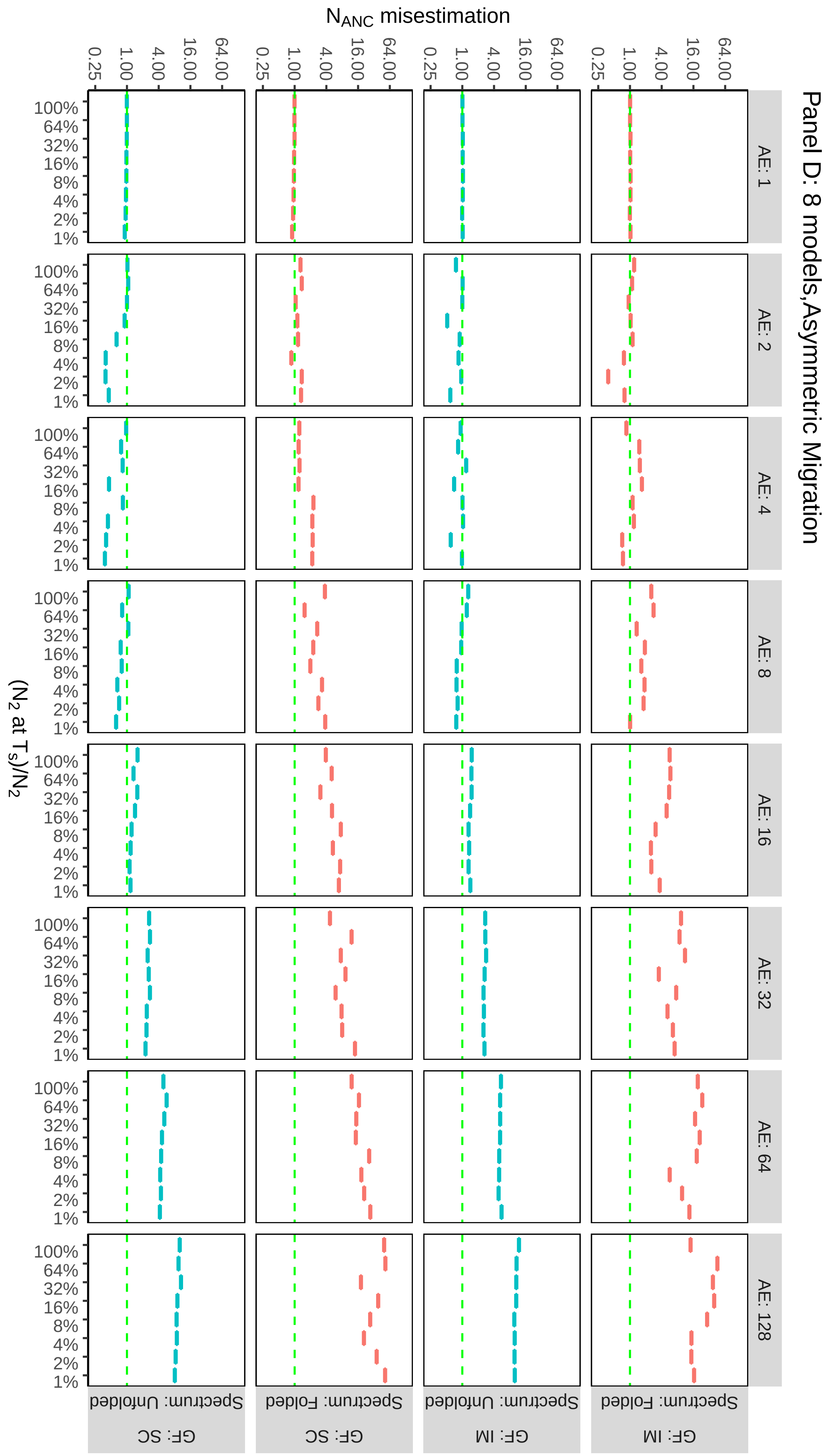
Panel D: 8 models,Asymmetric Migration
NANC misestimation
(N2 at Ts)/N2
AE: 1
AE: 2
AE: 4
AE: 8
AE: 16
AE: 32
AE: 64
AE: 128
Spectrum: Folded
Spectrum: Unfolded
GF: IM
GF: SC
0.25
1.00
4.00
16.00
64.00
100%
64%
32%
16%
8%
4%
2%
1%

Supplementary Fig. 14: misestimation of m_{12}. Columns is the grids group by simulation Ancestral Expansion (from 1, i.e. no expansion, to 128 x). The x axes represent the size of population two (N_2) at time of split (T_S) as a proportion of current (N_2). Rows of the plot grids group by gene flow scenario of the simulation (IM and SC) and by whether the unfolded or folded spectrum was used for inference. Panels **A** and **B** show m_{12} misestimation when the basic IM and SC models are used for inference, and under a symmetric and asymmetric migration scenario respectively. Panels **C** and **D** show m_{12} misestimation when the full 8 models (IM,SC,IM_{AE}, SC_{AE}, IM_B, SC_B, IM_{AEB}, SC_{AEB} are used. For each model, m_{12} misestimation was calculated as $m_{12}^{model}/m_{12}^{simulation}$

Panel A: IM−SC Models, Symmetric Migration

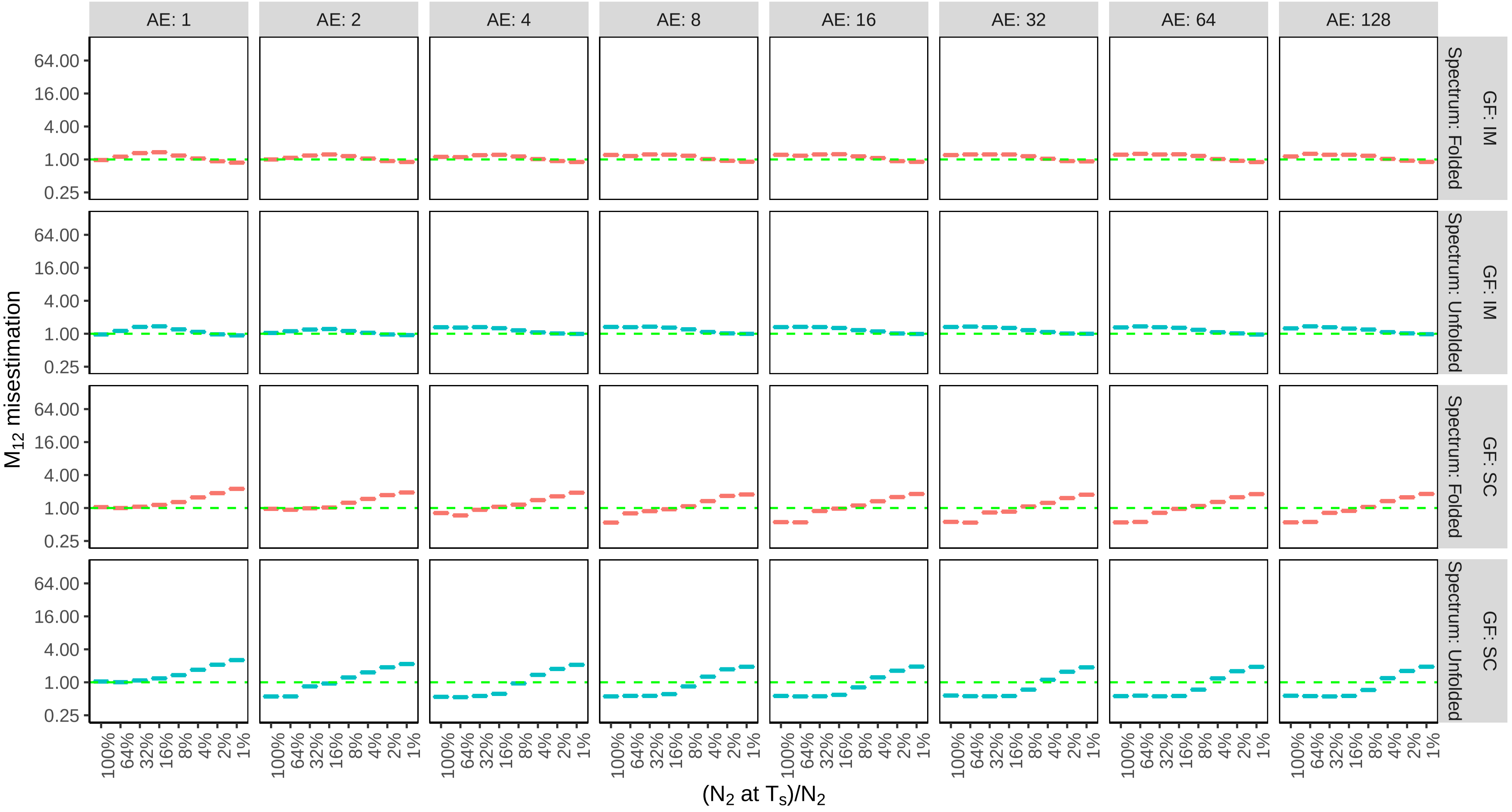

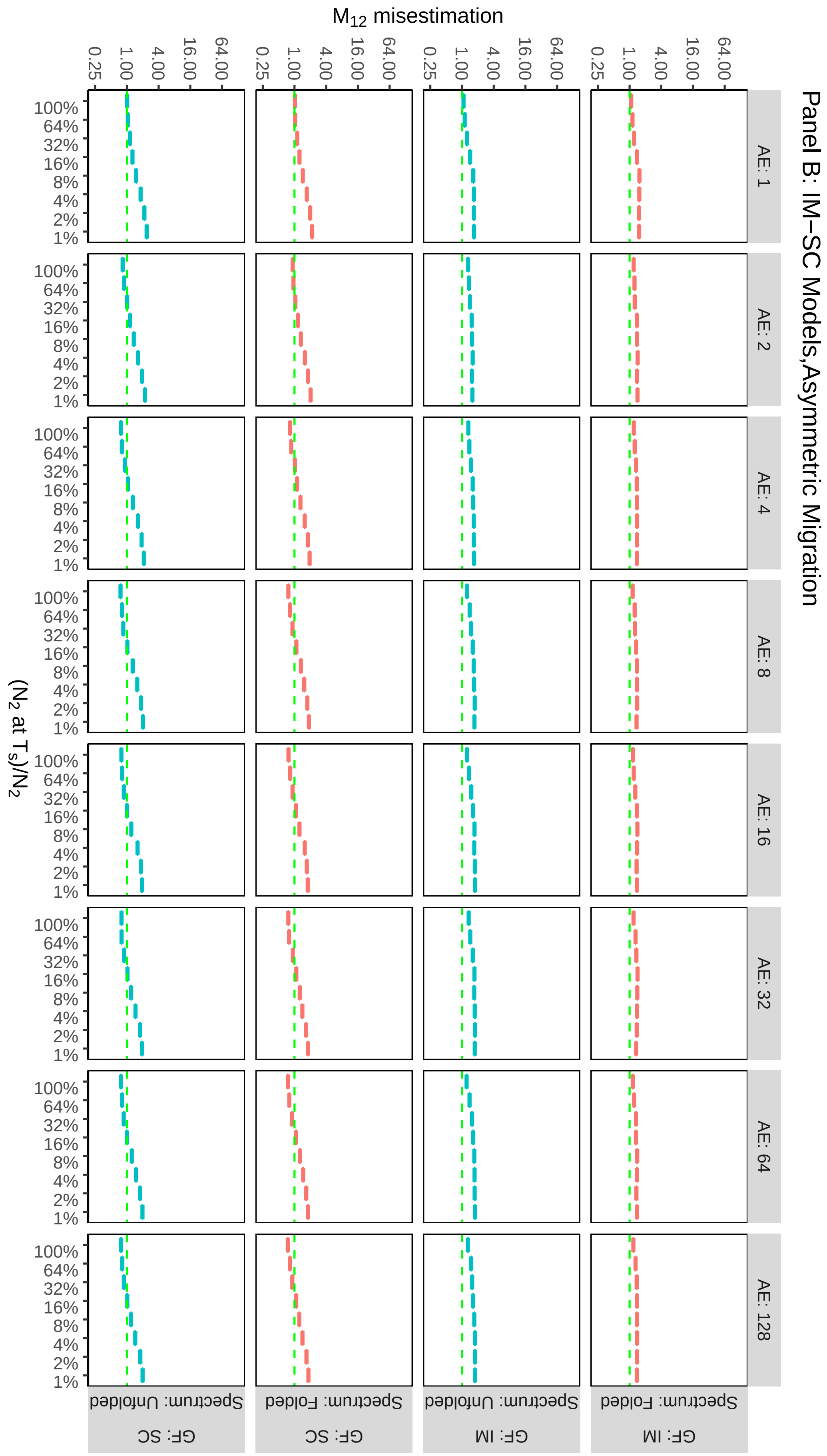

Panel B: IM–SC Models,Asymmetric Migration
M12 misestimation
0.25
1.00
4.00
16.00
64.00
AE: 1
AE: 2
AE: 4
AE: 8
AE: 16
AE: 32
AE: 64
AE: 128
100%
64%
32%
16%
8%
4%
2%
1%
(N2 at Ts)/N2
Spectrum: Unfolded
Spectrum: Folded
GF: SC
GF: IM

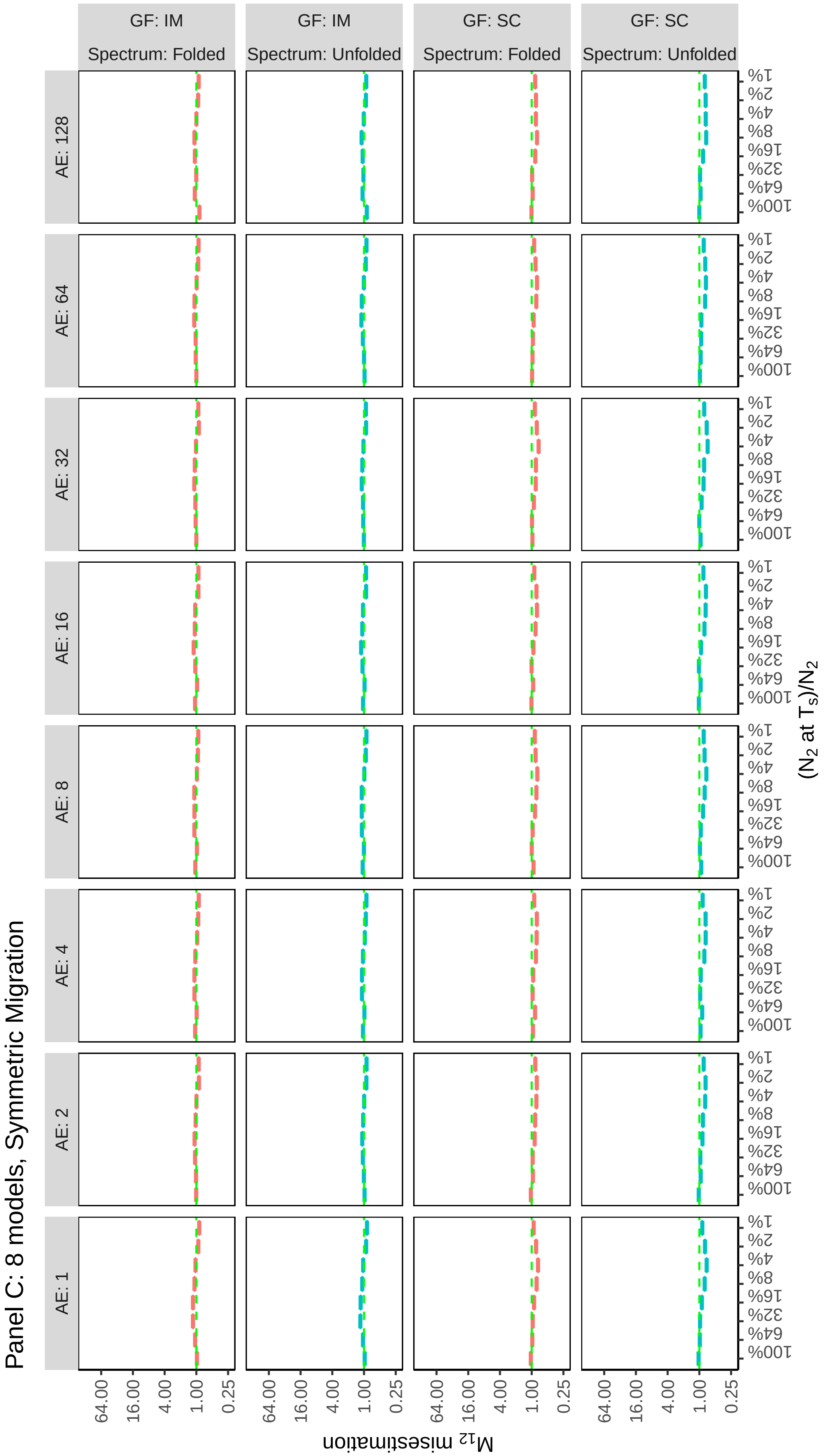
Panel C: 8 models, Symmetric Migration
GF: IM
Spectrum: Folded
GF: IM
Spectrum: Unfolded
GF: SC
Spectrum: Folded
GF: SC
Spectrum: Unfolded
AE: 128
AE: 64
AE: 32
AE: 16
AE: 8
AE: 4
AE: 2
AE: 1
1%
2%
4%
8%
16%
32%
64%
100%
(N2 at TS)/N2
64.00
16.00
4.00
1.00
0.25
M12 misestimation

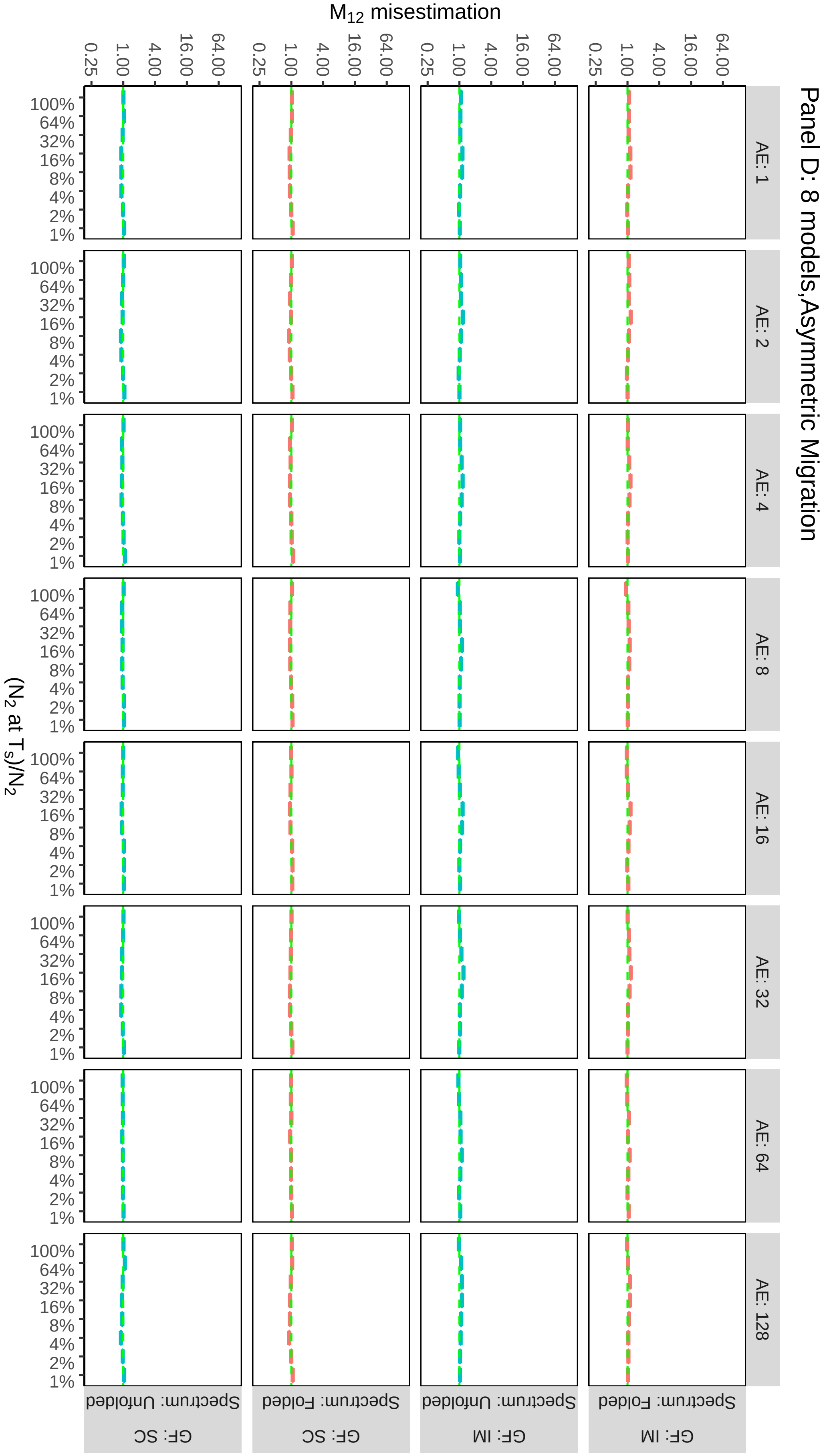
Panel D: 8 models,Asymmetric Migration
M12 misestimation
0.25
1.00
4.00
16.00
64.00
AE: 1
AE: 2
AE: 4
AE: 8
AE: 16
AE: 32
AE: 64
AE: 128
100%
64%
32%
16%
8%
4%
2%
1%
(N2 at Ts)/N2
Spectrum: Folded
GF: IM
Spectrum: Unfolded
GF: IM
Spectrum: Folded
GF: SC
Spectrum: Unfolded
GF: SC

Supplementary Fig. 15: misestimation of m_{21}. Columns is the grids group by simulation Ancestral Expansion (from 1, i.e. no expansion, to 128 x). The x axes represent the size of population two (N_2) at time of split (T_S) as a proportion of current (N_2). Rows of the plot grids group by gene flow scenario of the simulation (IM and SC) and by whether the unfolded or folded spectrum was used for inference. Panels **A** and **B** show m_{21} misestimation when the basic IM and SC models are used for inference, and under a symmetric and asymmetric migration scenario respectively. Panels **C** and **D** show (N_2) misestimation when the full 8 models (IM,SC,IM_{AE}, SC_{AE}, IM_B, SC_B, IM_{AEB}, SC_{AEB} are used. For each model, m_{21} misestimation was calculated as $m_{21}^{model}/m_{21}^{simulation}$

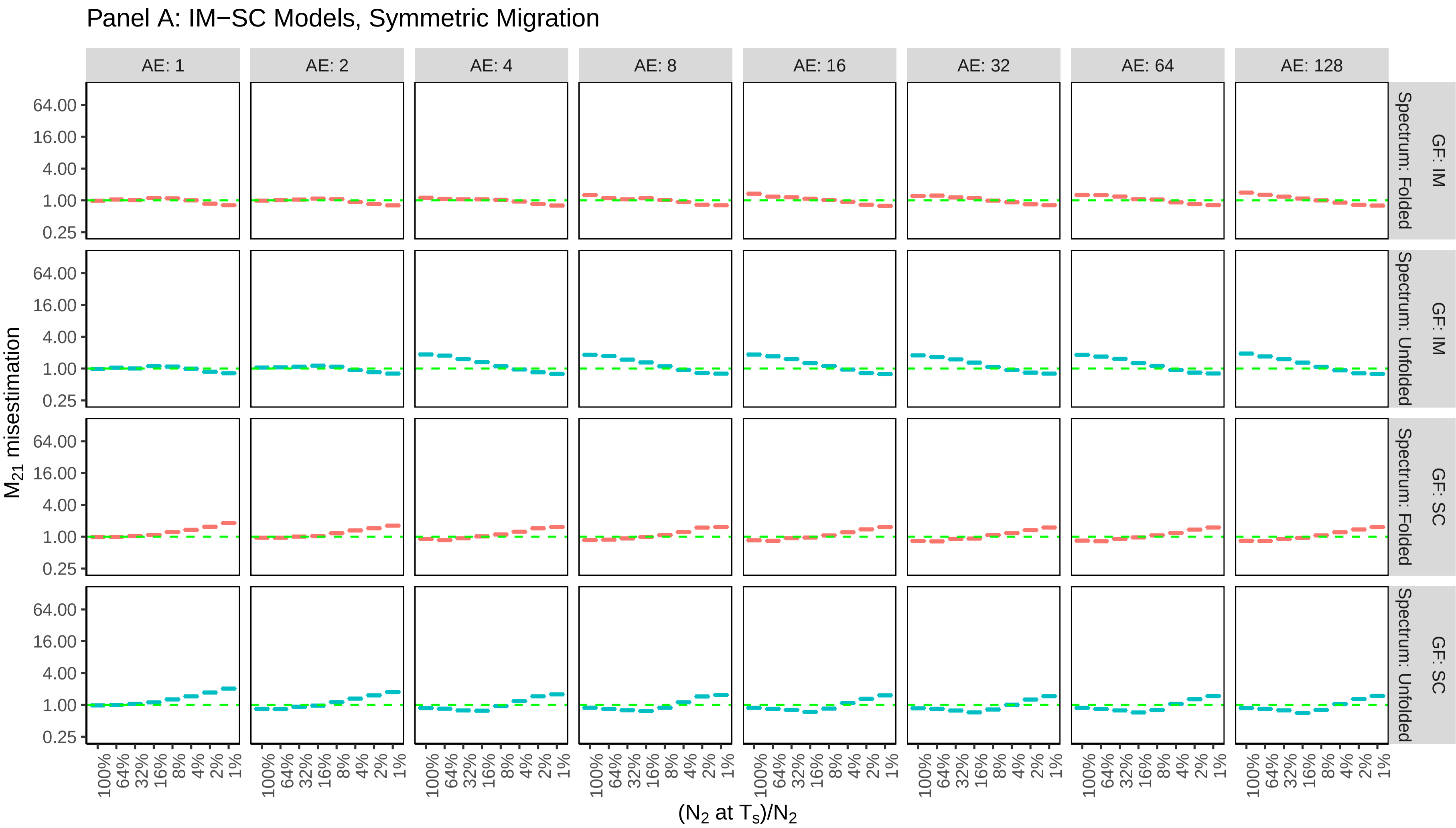

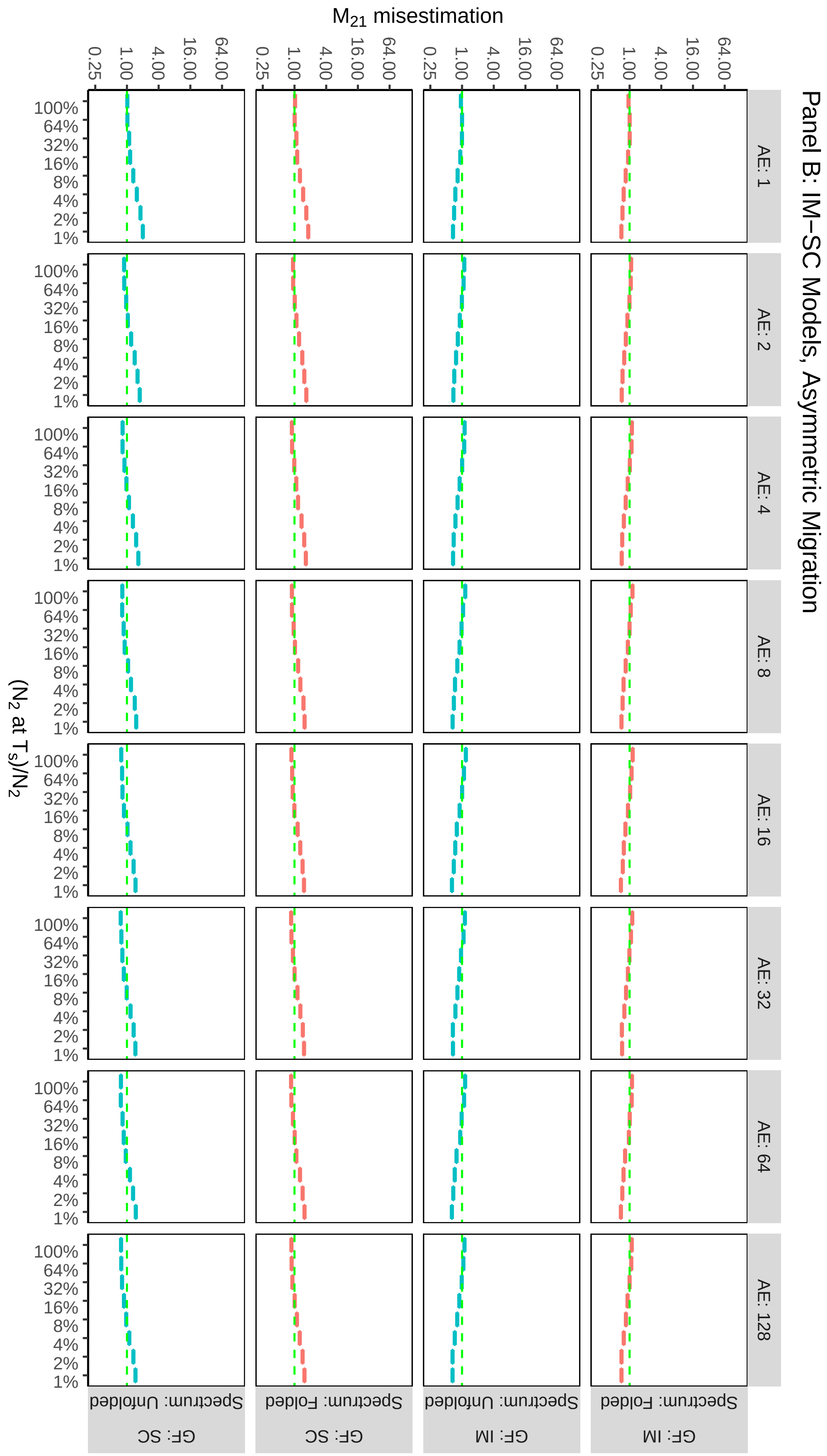
Panel B: IM–SC Models, Asymmetric Migration
M_{21} misestimation
$(N_2$ at $T_S)/N_2$
AE: 1
AE: 2
AE: 4
AE: 8
AE: 16
AE: 32
AE: 64
AE: 128
GF: IM
Spectrum: Folded
GF: IM
Spectrum: Unfolded
GF: SC
Spectrum: Folded
GF: SC
Spectrum: Unfolded
0.25
1.00
4.00
16.00
64.00
100%
64%
32%
16%
8%
4%
2%
1%

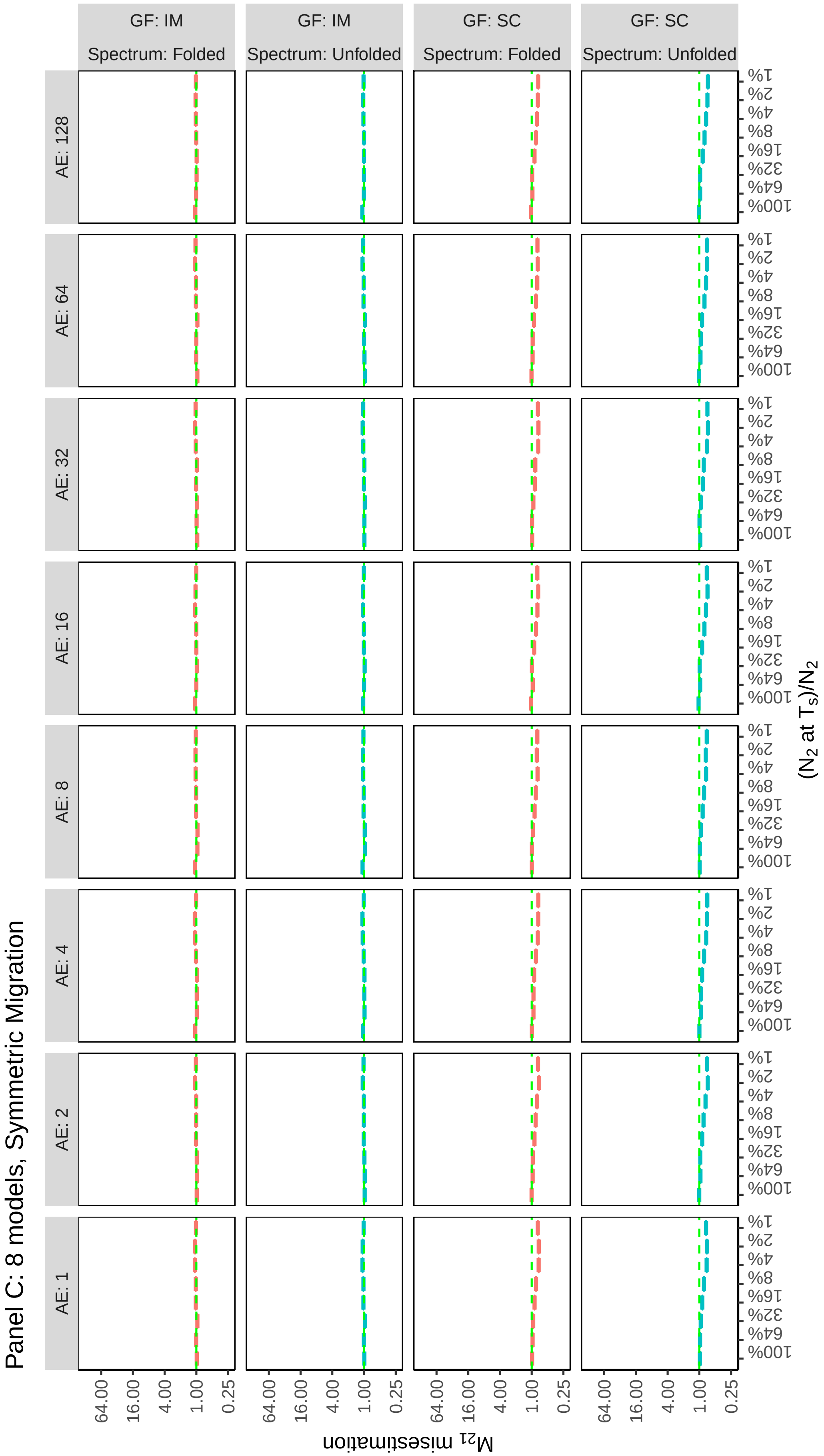
Panel C: 8 models, Symmetric Migration
GF: IM
Spectrum: Folded
GF: IM
Spectrum: Unfolded
GF: SC
Spectrum: Folded
GF: SC
Spectrum: Unfolded
AE: 1
AE: 2
AE: 4
AE: 8
AE: 16
AE: 32
AE: 64
AE: 128
64.00
16.00
4.00
1.00
0.25
M21 misestimation
1%
2%
4%
8%
16%
32%
64%
100%
(N2 at TS)/N2

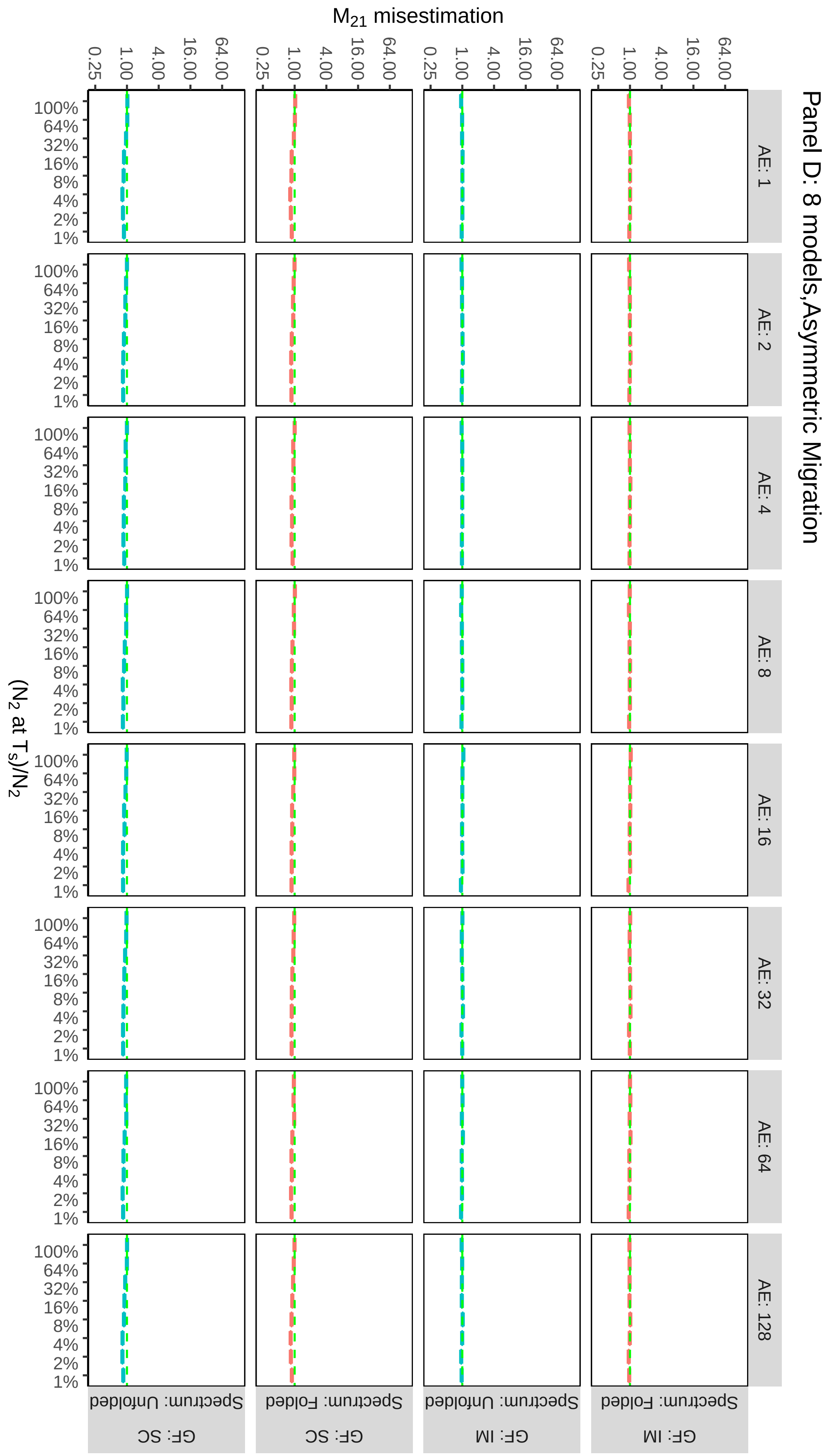
Panel D: 8 models,Asymmetric Migration
M21 misestimation
0.25
1.00
4.00
16.00
64.00
(N2 at Ts)/N2
100%
64%
32%
16%
8%
4%
2%
1%
AE: 1
AE: 2
AE: 4
AE: 8
AE: 16
AE: 32
AE: 64
AE: 128
GF: IM
Spectrum: Folded
GF: IM
Spectrum: Unfolded
GF: SC
Spectrum: Folded
GF: SC
Spectrum: Unfolded

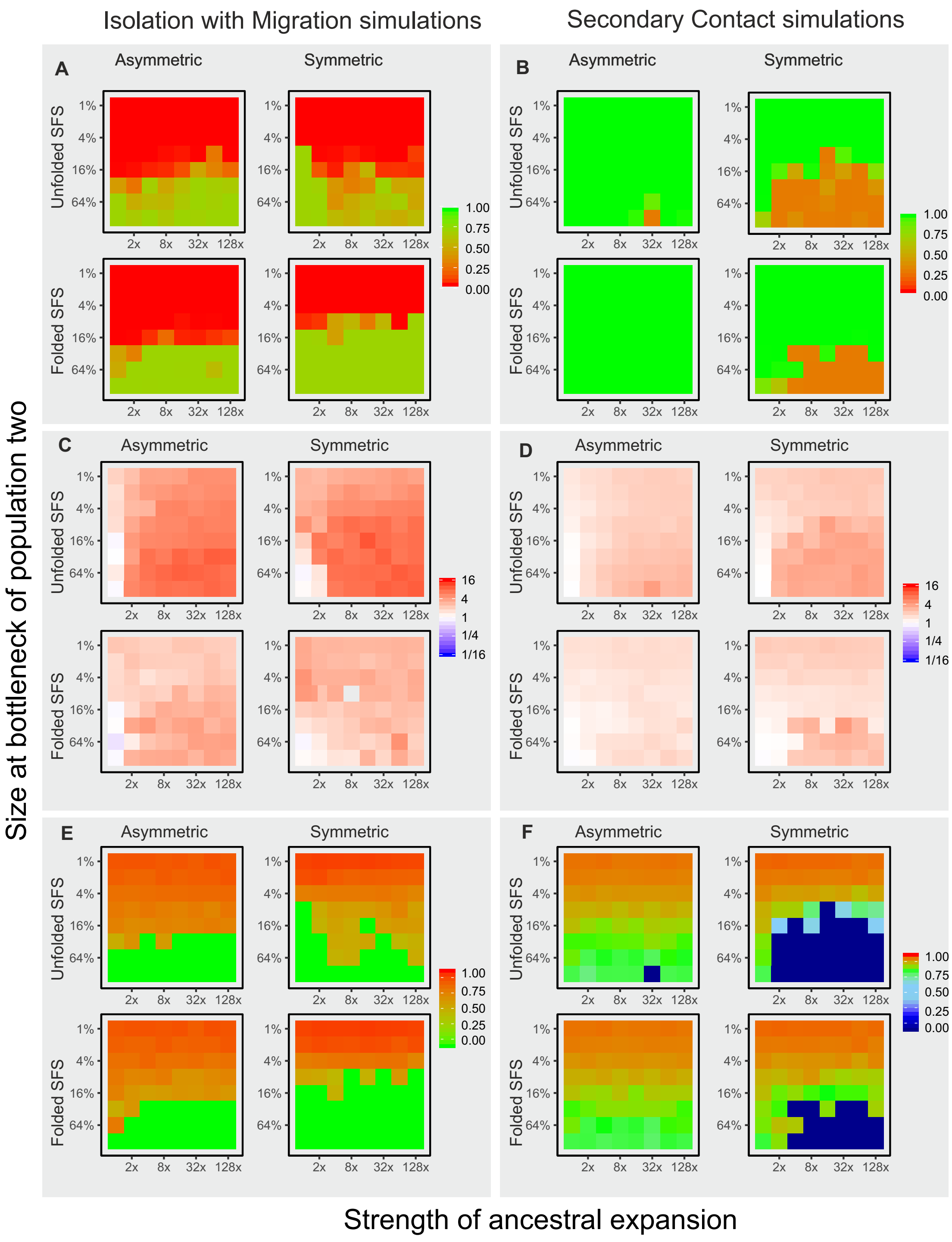

Supplementary Fig. 16: Model choice and parameter misestimation for the simple IM-SC models for all simulations of the smaller data sets (100 000 loci). Left panels (**A, C, E**) show results from simulations with constant migration (IM), right panels (**B, D, F**) show results from simulation with a period of strict isolation (SC). Within each panel, results are shown for simulations with symmetric and asymmetric migration, and for model estimates using the folded or unfolded jAFS. Within each panel, each graph represents the values for all 64 simulations as per Fig. 1. Panel **A** & **B** show weight of evidence for the correct gene flow scenario (0-1). Panel **C** & **D** show misestimation of the parameter T_S as T_S model / T_S simulation. Panels **E** and **F** show the estimated proportion of the divergence time for which the model inferred strict isolation (green represents the correct time, i.e. 0 for IM model and 0.75 for SC models). This figure is the equivalent of Fig. 2 in the main manuscript, but representing values for the smaller simulated datatsets.

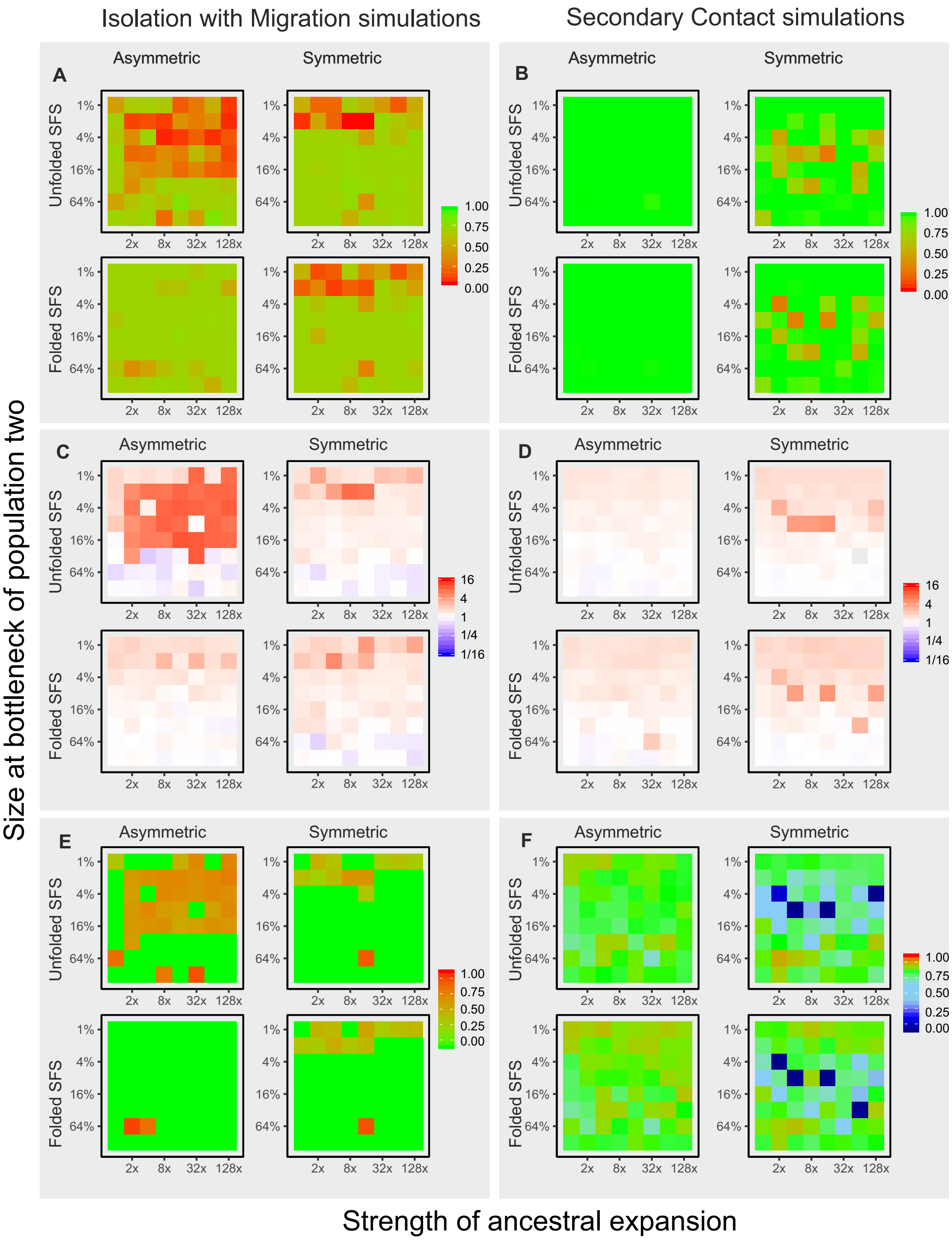

Supplementary Fig. 17: Model choice and parameter misestimation for all 8 divergence models for all simulations of the smaller data sets (100 000 loci). Left panels (**A, C, E**) show results from simulations with constant migration (IM), left panels (**B, D, F**) show results from simulation with a period of strict isolation (SC). Within each panel, results are shown for simulations with symmetric and asymmetric migration, and for model estimates using the folded or unfolded jAFS. Within each panel, each graph represents the values for all 64 simulations as per Fig. 1. Panel **A** & **B** show weight of evidence for the correct gene flow scenario (0-1). Panel **C** & **D** show misestimation of the parameter T_S as T_S model / T_S simulation). Panels **E** and **F** show the estimated proportion of the divergence time for which the model inferred strict isolation (green represents the correct time, i.e. 0 for IM model and 0.75 for SC models). This figure is the equivalent of Supplementary Fig. 2, but representing values for the smaller simulated data sets.

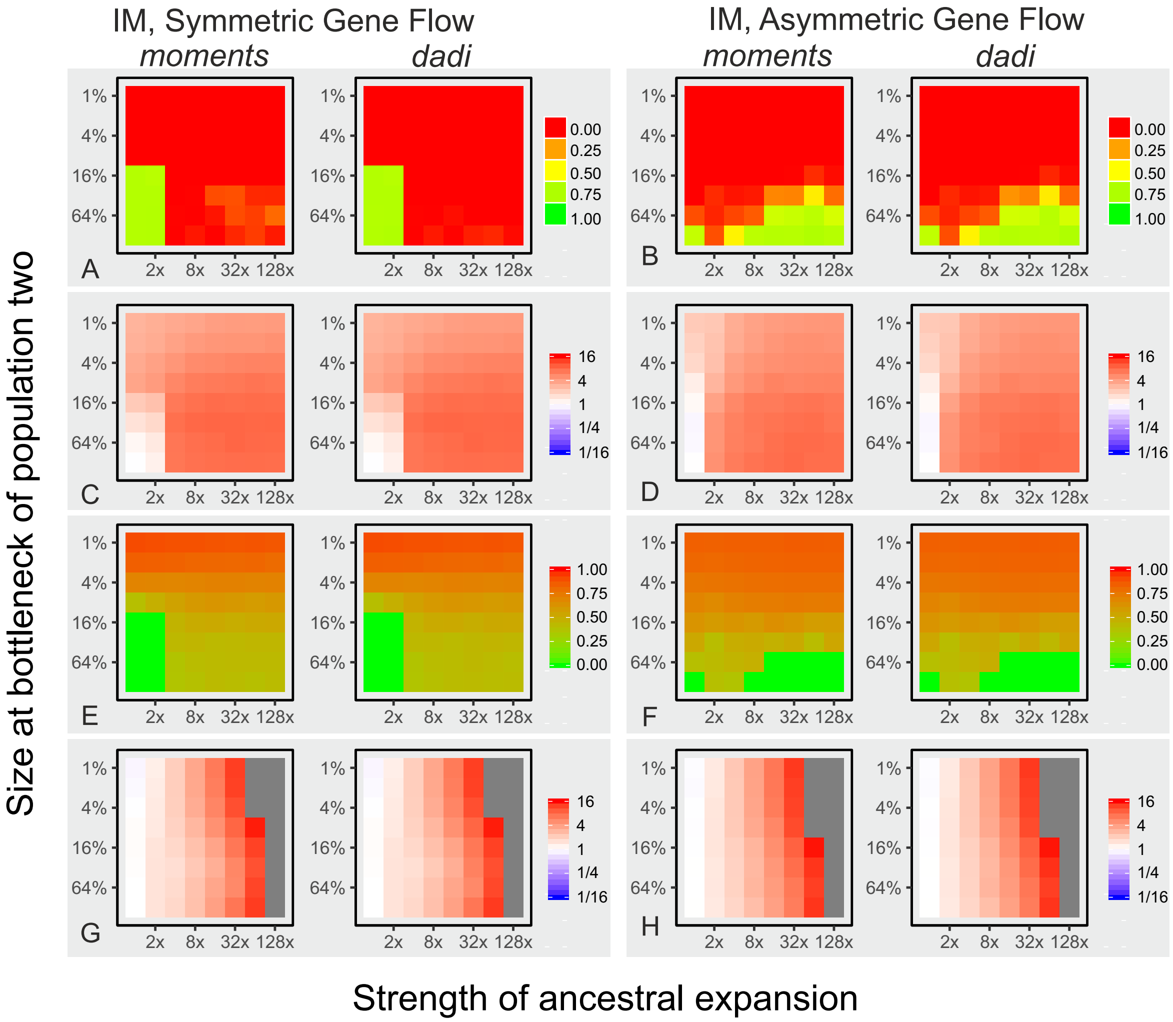

Supplementary Fig. 18: Comparison of model choice and parameter estimates of the IM and SC simple models in *moments* and *dadi* for IM simulations (1 million loci) under a scenario of symmetric (left panels: **A**, **C**, **E**, **G**) and asymmetric (right panels: **B**,**D**,**F**,**H**) gene flow. Panel **A** and **B** show weight of evidence for the correct gene flow scenario (0-1). Panel **C** and **D** show misesistemation of the parameter T_S (calculated as T_S model / T_S simulation) for the best fitting model. Panels **E** and **F** show the estimated proportion of the divergence time for which the best fitting model inferred strict isolation (green represent the correct time, i.e. 0 for IM model). Panels **G** and **H** show the misestimaton of the ancestral population size N_{ANC} (N_{ANC} model / N_{ANC} simulations). Notice that *moments* and *dadi* gave nearly identical results. Grey squares represent off-scale values.

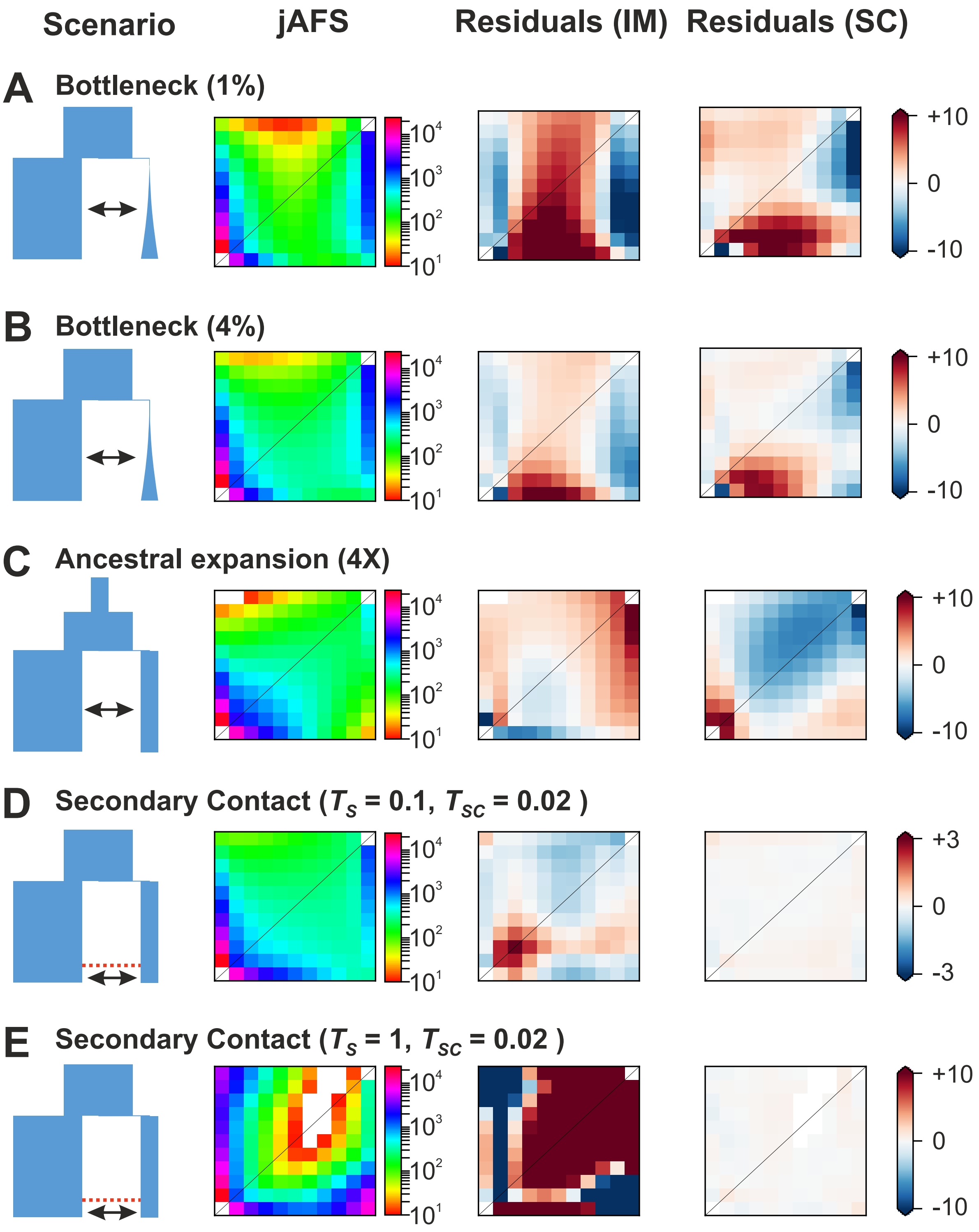

Supplementary Fig. 19: Examples of residual plots from optimized IM and SC models under a range of different demographic scenarios. All scenarios, with the exception of panel **D**, come from the recent divergence scenarios depicted in Fig. 1B. $T_S = 0.05$ for IM scenarios (**A,B,C**) and $T_S = 0.1$ for the SC scenario in **C**. In panel **D** we show an example of a SC scenario with a long period of strict isolation ($T_S = 1$), all other parameters are as per other recent divergence scenario. For each panel we show the demographic scenario (left, population sizes are in scale) and the observed jAFS from the simulations (jAFS), projected down to 10 alleles for ease of visualization. We then show the residual plots from the optimized IM and SC models. In all scenarios, SC showed strong statistical support. Indeed, SC models fit the data considerably better, not only when the data were simulated under a SC scenario (**D & E**), but only when there was a strong **A** or mild **B** unaccounted bottleneck, and to a lesser degree when there was an unaccounted ancestral expansion **C**.

Supplementary Fig. 20: Genomic landscape of differentiation between North Sea and Baltic Sea turbots across all chromosomes. The lines represent patterns of genetic diversity calculated in non-overlapping windows of 250 kb. The red line represents π in the Baltic Sea, the blue line π in the North Sea, and the black line represents d_{xy} . Dots along the chromosomes represent q values (FDR) on a negative log scales from fastPCA genome scan for selection for each individual SNP, the dotted line remarks the 0.1 FDR and green dots represents significant outliers according to the HHM test.

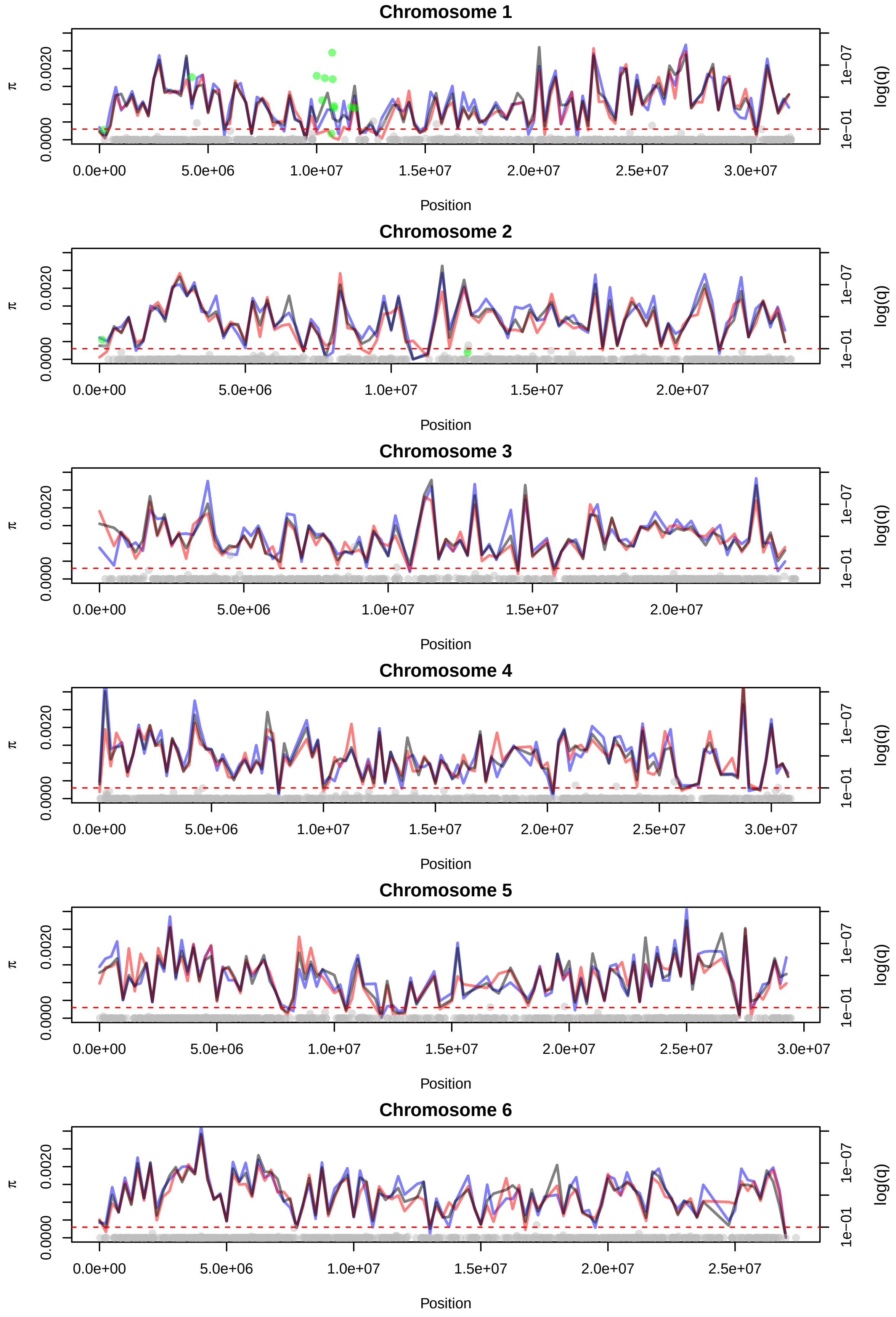

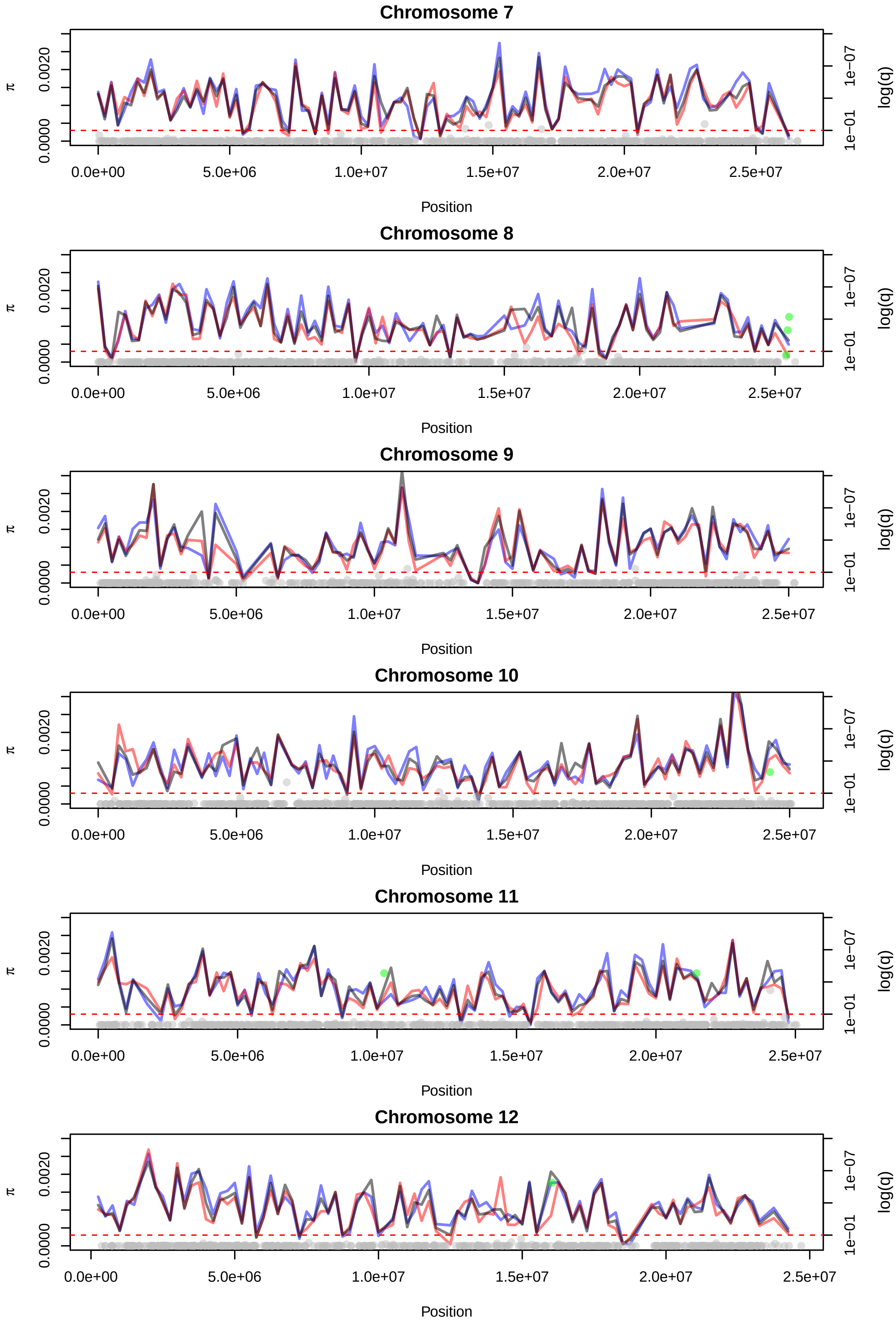
Chromosome 7
π
0.0000
0.0020
1e−01
1e−07
log(q)
0.0e+00
5.0e+06
1.0e+07
1.5e+07
2.0e+07
2.5e+07
Position
Chromosome 8
π
0.0000
0.0020
1e−01
1e−07
log(q)
0.0e+00
5.0e+06
1.0e+07
1.5e+07
2.0e+07
2.5e+07
Position
Chromosome 9
π
0.0000
0.0020
1e−01
1e−07
log(q)
0.0e+00
5.0e+06
1.0e+07
1.5e+07
2.0e+07
2.5e+07
Position
Chromosome 10
π
0.0000
0.0020
1e−01
1e−07
log(q)
0.0e+00
5.0e+06
1.0e+07
1.5e+07
2.0e+07
2.5e+07
Position
Chromosome 11
π
0.0000
0.0020
1e−01
1e−07
log(q)
0.0e+00
5.0e+06
1.0e+07
1.5e+07
2.0e+07
2.5e+07
Position
Chromosome 12
π
0.0000
0.0020
1e−01
1e−07
log(q)
0.0e+00
5.0e+06
1.0e+07
1.5e+07
2.0e+07
2.5e+07
Position

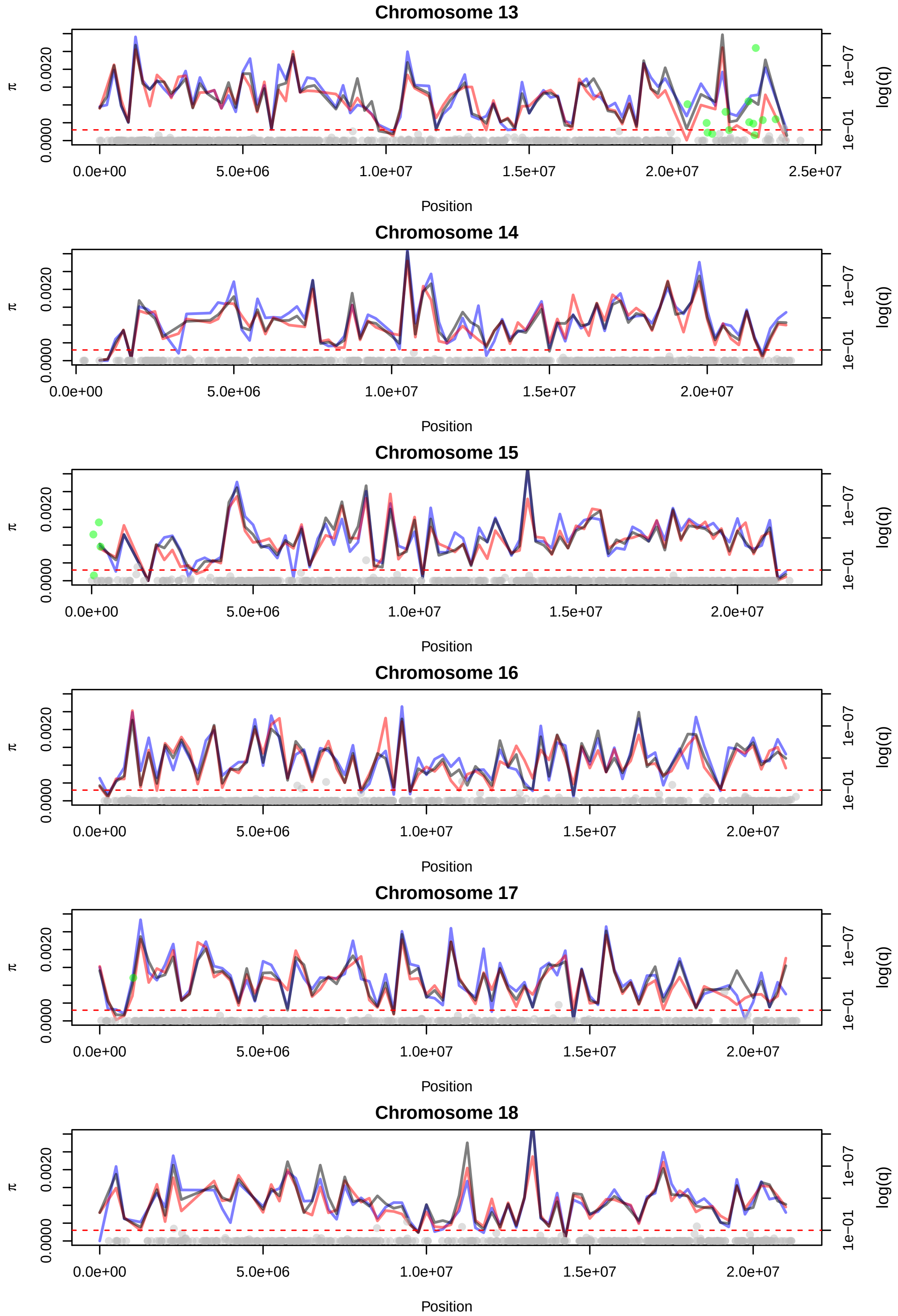
Chromosome 13
Chromosome 14
Chromosome 15
Chromosome 16
Chromosome 17
Chromosome 18
π
0.0000
0.0020
log(q)
1e−01
1e−07
Position
0.0e+00
5.0e+06
1.0e+07
1.5e+07
2.0e+07
2.5e+07

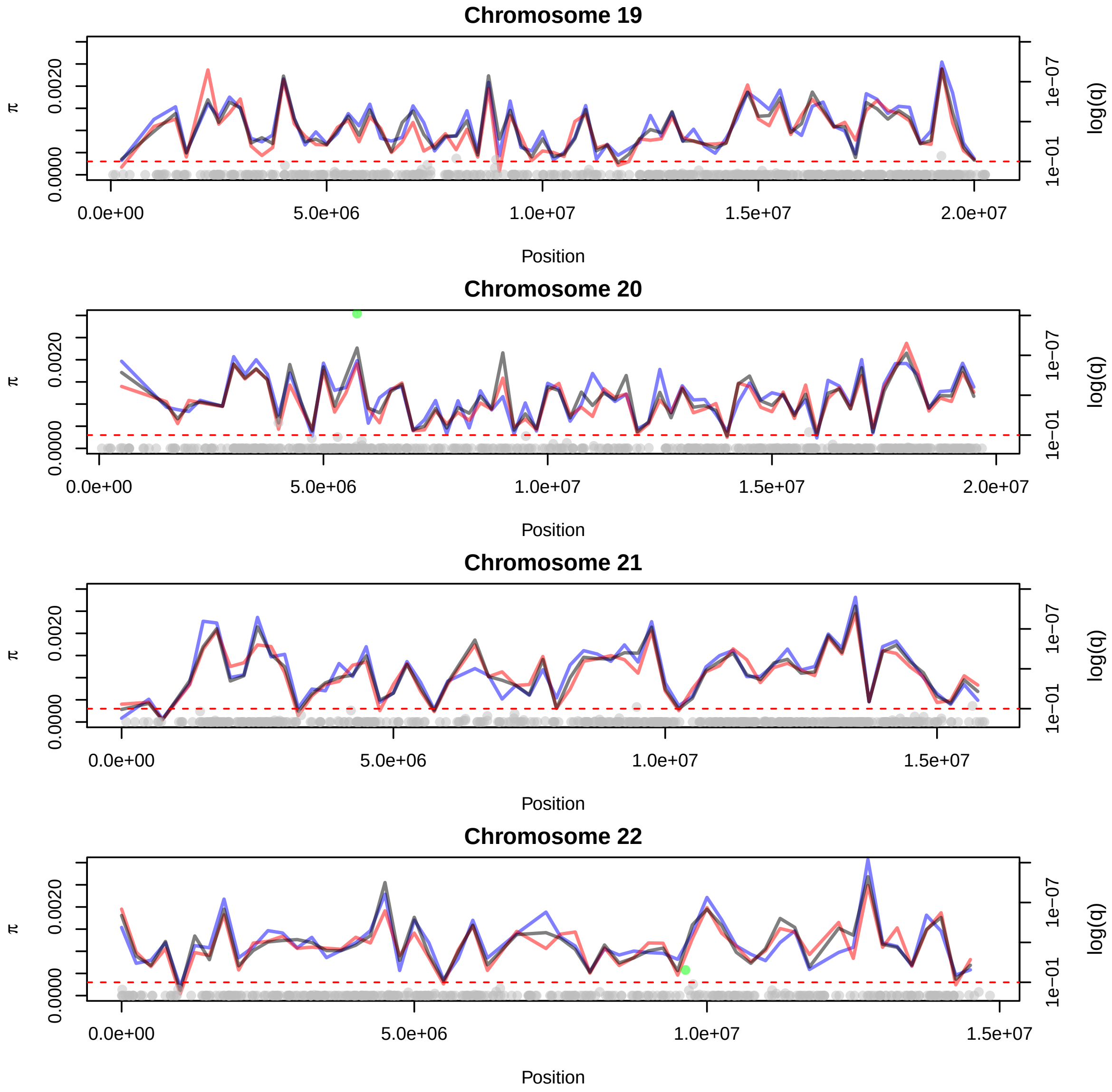
Chromosome 19
π
0.0000
0.0020
1e−01
1e−07
log(q)
0.0e+00
5.0e+06
1.0e+07
1.5e+07
2.0e+07
Position
Chromosome 20
π
0.0000
0.0020
1e−01
1e−07
log(q)
0.0e+00
5.0e+06
1.0e+07
1.5e+07
2.0e+07
Position
Chromosome 21
π
0.0000
0.0020
1e−01
1e−07
log(q)
0.0e+00
5.0e+06
1.0e+07
1.5e+07
Position
Chromosome 22
π
0.0000
0.0020
1e−01
1e−07
log(q)
0.0e+00
5.0e+06
1.0e+07
1.5e+07
Position
